# Multiplex Targeted Proteomic Analysis of Cytokine Ratios for ICU Mortality in Severe COVID-19

**DOI:** 10.3390/proteomes13030035

**Published:** 2025-08-02

**Authors:** Rúben Araújo, Cristiana P. Von Rekowski, Tiago A. H. Fonseca, Cecília R. C. Calado, Luís Ramalhete, Luís Bento

**Affiliations:** 1 NMS—NOVA Medical School, FCM—Faculdade de Ciências Médicas, Universidade NOVA de Lisboa, Campo dos Mártires da Pátria 130, 1169-056 Lisbon, Portugal; rubenalexandredinisaraujo@gmail.com (R.A.);; 2 CHRC—Comprehensive Health Research Centre, Universidade NOVA de Lisboa, 1150-082 Lisbon, Portugal; 3 ISEL—Instituto Superior de Engenharia de Lisboa, Instituto Politécnico de Lisboa, Rua Conselheiro Emídio Navarro 1, 1959-007 Lisbon, Portugal; 4 Institute for Bioengineering and Biosciences (iBB), The Associate Laboratory Institute for Health and Bioeconomy-i4HB, Instituto Superior Técnico (IST), Universidade de Lisboa (UL), Av. Rovisco Pais, 1049-001 Lisbon, Portugal; 5 IPST—Instituto Português do Sangue e da Transplantação, Alameda das Linhas de Torres 117, 1769-001 Lisbon, Portugal; 6 iNOVA4Health—Advancing Precision Medicine, RG11: Reno-Vascular Diseases Group, NMS—NOVA Medical School, FCM—Faculdade de Ciências Médicas, Universidade NOVA de Lisboa, 1169-056 Lisbon, Portugal; 7 Intensive Care Department, ULSSJ—Unidade Local de Saúde São José, Rua José António Serrano, 1150-199 Lisbon, Portugal; 8 Integrated Pathophysiological Mechanisms, CHRC—Comprehensive Health Research Centre, NMS—NOVA Medical School, FCM—Faculdade de Ciências Médicas, Universidade NOVA de Lisboa, Campo Mártires da Pátria 130, 1169-056 Lisbon, Portugal

**Keywords:** cytokine profiling, ICU mortality, proteomics, multiplex cytokine analysis, complex cytokine ratios, machine learning, critical care

## Abstract

Background: Accurate and timely prediction of mortality in intensive care unit (ICU) patients, particularly those with COVID-19, remains clinically challenging due to complex immune responses. Proteomic cytokine profiling holds promise for refining mortality risk assessment. Methods: Serum samples from 89 ICU patients (55 discharged, 34 deceased) were analyzed using a multiplex 21-cytokine panel. Samples were stratified into three groups based on time from collection to outcome: ≤48 h (Group 1: Early), >48 h to ≤7 days (Group 2: Intermediate), and >7 days to ≤14 days (Group 3: Late). Cytokine levels, simple cytokine ratios, and previously unexplored complex ratios between pro- and anti-inflammatory cytokines were evaluated. Machine learning-based feature selection identified the most predictive ratios, with performance evaluated by area under the curve (AUC), sensitivity, and specificity. Results: Complex cytokine ratios demonstrated superior predictive accuracy compared to traditional severity markers (APACHE II, SAPS II, SOFA), individual cytokines, and simple ratios, effectively distinguishing discharged from deceased patients across all groups (AUC: 0.918–1.000; sensitivity: 0.826–1.000; specificity: 0.775–0.900). Conclusions: Multiplex cytokine profiling enhanced by computationally derived complex ratios may offer robust predictive capabilities for ICU mortality risk stratification, serving as a valuable tool for personalized prognosis in critical care.

## 1. Introduction

Mortality in intensive care units (ICUs) remains a persistent and complex challenge, stemming from both the heterogeneity of critically ill patients and the high prevalence of comorbidities. Accurate early assessment of a patient’s risk of death is essential for guiding clinical decision-making, allocating scarce resources, and ultimately improving patient outcomes [1]. The emergence of the Coronavirus Disease 2019 (COVID-19), caused by the severe acute respiratory syndrome coronavirus 2 (SARS-CoV-2), has compounded these difficulties by exacerbating inherent strains in critical care systems nationwide and worldwide [2,3]. Critically ill COVID-19 patients often exhibit profound immune dysregulation, overlapping with the pathophysiology of other acute disorders such as sepsis and acute respiratory distress syndrome (ARDS) [4,5]. These overlapping features blur the clinical picture and limit the effectiveness of conventional diagnostic and prognostic methods. As a result, there is an urgent need for more nuanced, dynamic, and biologically informed strategies to project patient trajectories and mortality risk in this highly volatile setting [6,7]. Notably, a large ancillary study demonstrated that 28-day mortality approximates ICU mortality in moderate-to-severe ARDS, suggesting it may serve as a practical substitute outcome [8].

Several widely used scoring systems—Acute Physiology and Chronic Health Evaluation II (APACHE II) [9], Simplified Acute Physiology Score (SAPS) [10], and Sequential Organ Failure Assessment (SOFA) [11]—each serves distinct purposes in critical care. Importantly, APACHE II and SAPS were originally developed to estimate cumulative hospital mortality, including deaths both during the ICU stay and after transfer to the infirmary.

APACHE II is primarily used at admission and incorporates acute physiological variables (e.g., temperature, arterial pH, serum creatinine) alongside chronic health factors (e.g., a history of severe organ dysfunction) to estimate hospital mortality. Recent findings suggest that its predictive utility may improve when used dynamically; for example, APACHE II measured on day 3 of ICU stay was found to more accurately predict hospital mortality than at admission [12]. Nonetheless, the validity of such predictions depends heavily on proper statistical calibration, such as the Hosmer–Lemeshow test, and on applying the score at the right time point. Additionally, small shifts in key variables—like blood pH, body temperature, or FiO_2_—can significantly alter mortality risk at extreme values, though this is often masked by conventional threshold approaches [13].

The SAPS scoring system was initially designed to streamline computation and has since evolved into SAPS II and SAPS 3, with the latter introducing customized equations tailored to major geographic regions to enhance calibration [14]. Yet, its performance can still vary substantially by institution and population; a European multicenter study found that both SAPS II and SAPS 3 showed miscalibration influenced by patient- and center-level characteristics, with some variability remaining unexplained [15]. In targeted applications such as sepsis, SAPS 3 has outperformed SAPS II, SOFA, SIRS (Systemic Inflammatory Response Syndrome), and OASIS (Oxford Acute Severity of Illness Score) for predicting 28-day mortality [16]. A separate study also showed that a customized version of SAPS 3 (c-SAPS 3) offered superior calibration compared to APACHE II, SAPS II, and standard SAPS 3, further emphasizing the value of local or population-specific adaptation [17].

In contrast to APACHE II and SAPS, which are designed to predict overall hospital mortality, SOFA was specifically developed for continuous organ dysfunction monitoring in the ICU. By tracking daily changes in six organ systems—cardiovascular, respiratory, hepatic, renal, hematologic, and neurological—SOFA provides a dynamic, longitudinal picture of patient trajectories rather than a single early estimate. However, the score is now nearly three decades old, and recent discussions have highlighted its limitations in the face of evolving ICU practices, with calls to modernize SOFA including the proposal of new conceptual updates [18].

Nevertheless, despite their widespread adoption, none of these systems incorporates direct measures of immune status—such as cytokine profiles—potentially overlooking a critical dimension of ICU pathophysiology. Moreover, the overwhelming majority of published research evaluating ICU mortality relies on these standard indices, leaving the role of cytokine-based biomarkers in mortality discrimination or prediction comparatively underexplored. Furthermore, studies that have applied SOFA to COVID-19 patients have reported inconsistent results, with many indicating area under the curve (AUC) values in the high 60s to low 70s, suggesting only moderate predictive utility [19,20]. These findings underscore the need for more sophisticated predictive models that incorporate changes in patient status, disease-specific biomarkers, and a broader range of host responses. To that end, modern machine learning approaches such as the Super ICU Learner Algorithm (SICULA) have already demonstrated values for AUC as high as 0.88–0.94 and better calibration than APACHE II, SAPS II, or SOFA [21], further illustrating how advanced analytics could reshape outcome prediction.

Cytokines have emerged as potent biomarkers for mortality risk stratification in critically ill patients [22]. These small protein mediators, released upon infection, trauma, or systemic inflammation, play vital roles in modulating innate and adaptive immune pathways, offering a real-time snapshot of the host inflammatory state [23,24]. Cytokines of particular interest include interleukin-6 (IL-6), tumor necrosis factor-alpha (TNF-α), interleukin-8 (IL-8), and interleukin-10 (IL-10), each having been linked to mortality in conditions such as acute cardiac events [25], renal failure [26], sepsis [27], ARDS [28,29], and notably COVID-19 [30]. Elevated levels of IL-6, for example, are commonly associated with poor outcomes and may underlie the “cytokine storm” syndrome observed in severe COVID-19 cases. Similarly, increased concentrations of TNF-α and IL-8 have been correlated with severe systemic inflammation and multi-organ failure [31], whereas high IL-10—despite its anti-inflammatory function—may reflect an insufficient compensatory mechanism for controlling overwhelming inflammation [32]. Like traditional clinical scoring systems such as APACHE, Glasgow Coma Scale (GCS), and SOFA, individual cytokines—while widely used as indicators of disease severity—can fall short when interpreted in isolation, with performance metrics such as AUC often not exceeding the low 70s [33].

The balance between pro-inflammatory and anti-inflammatory cytokines is a pivotal determinant of outcomes in ICU patients [34]. This equilibrium is especially tenuous in COVID-19, where unrestrained immune activation can rapidly swing from excessive inflammation to immunosuppression, both of which can be lethal [35,36,37,38]. Pro-inflammatory mediators such as IL-6, Interleukin-1 beta (IL-1β), TNF-α, interferon-gamma (IFN-γ), and Interleukin-17A (IL-17A), though central to pathogen clearance, can drive tissue injury, vascular compromise, and systemic complications—including ARDS and coagulopathy—when overproduced [39,40,41,42]. Conversely, anti-inflammatory cytokines such as IL-10, Interleukin-4 (IL-4), Interleukin-13 (IL-13), and Interleukin-21 (IL-21) help mitigate tissue damage and prevent autoimmunity, yet their overexpression in critically ill patients has been linked to immune exhaustion or “immunoparalysis”, which predisposes individuals to secondary infections and increased mortality [39,43,44,45,46]. In COVID-19, this precarious interplay is frequently marked by both excessive pro-inflammatory cytokine release and simultaneous anti-inflammatory signaling, creating a discordant immune state that complicates patient management [47,48]. Quantifying this complex cytokine environment may therefore significantly improve mortality prediction in ICU populations.

Given this background, this study addresses the gap in ICU mortality prediction for COVID-19 patients by coupling cytokine profiling with computational modeling. A total of 125 serum samples were collected from 89 critically ill patients admitted to a tertiary ICU, who were then categorized into three outcome-based groups according to time from blood collection to either discharge or death in the ICU: Group 1 (≤48 h), Group 2 (>48 h to ≤7 days), and Group 3 (>7 days to ≤14 days). Using a high-sensitivity multiplex assay, 21 cytokines were quantified, and more than 19,000 biologically relevant cytokine ratios were programmatically generated. The main objective was to determine whether complex cytokine ratios refined via entropy-based feature selection could match or surpass the predictive performance of established prognostic scores, individual cytokine measurements, and even more novel omics-based approaches [49] in forecasting mortality. Building on prior research into cytokine ratios for detecting infectious complications in COVID-19 patients [50], this approach extends that method to the broader and more critical endpoint of ICU mortality.

Ultimately, the overarching goal is to advance precision medicine in critical care by providing new evidence on the prognostic value of immune-based strategies for high-risk patient populations. This study thus contributes to a growing body of evidence suggesting that immune profiling, particularly cytokine-based analysis, may offer complementary or alternative approaches to traditional scoring systems.

## 2. Materials and Methods

### 2.1. Workflow Overview

**Figure 1:**
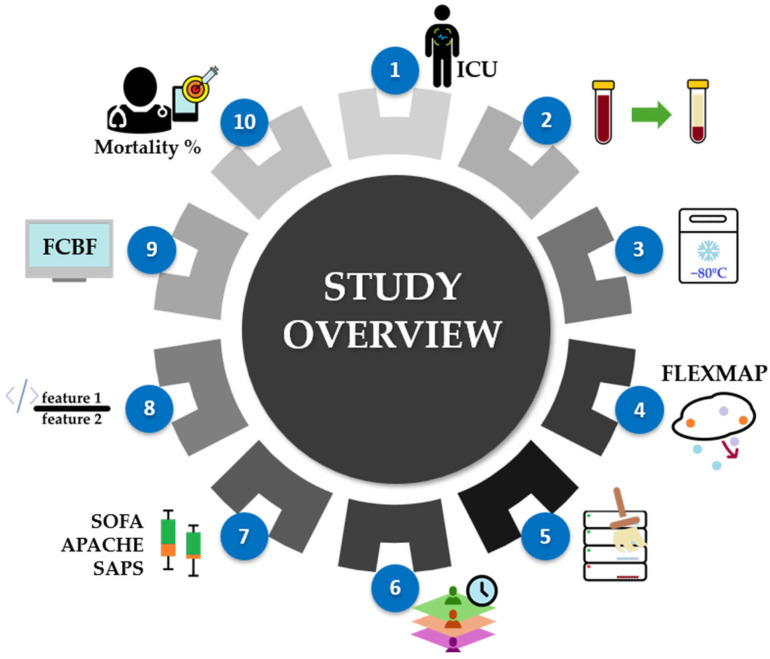
Study workflow overview. The process includes: (1) selection of ICU patients with confirmed COVID-19, (2) blood collection and serum separation, (3) sample preservation at −80 °C, (4) cytokine quantification via a high-sensitivity multiplex assay, (5) data transformation and quality control, (6) stratification of samples by outcome timing (≤48 h, >48 h to ≤7 days, >7 days to ≤14 days), (7) statistical evaluation incorporating clinical severity scores (SOFA, APACHE, SAPS), individual cytokine levels, and traditional ratios, (8) generation of complex cytokine ratios, (9) feature selection using Fast Correlation-Based Filter, and (10) supervised machine learning for mortality risk classification and biomarker discovery.

Figure 1 illustrates the overall workflow of the study, from patient enrollment through to model evaluation. The process begins with the selection of ICU patients with confirmed COVID-19 admitted to a tertiary hospital, followed by routine blood collection and serum separation, with samples promptly frozen at −80 °C to preserve cytokine integrity for downstream analysis. A high-sensitivity multiplex assay was used to quantify a panel of 21 pro- and anti-inflammatory cytokines. The resulting data then underwent extraction, transformation, and quality control steps to ensure consistency and reliability. Samples were then stratified based on the time between blood collection and ICU discharge or death, forming three outcome-based groups for both discharged and deceased patients. Statistical and computational pipelines, including univariate analysis, multivariate exploration, and feature selection algorithms, were applied to evaluate individual cytokines, standard ratios, and programmatically generated complex ratios. These features were subsequently used to train machine learning models aimed at discriminating between discharged and deceased ICU patients across the defined time windows.

### 2.2. Study Population

A total of 89 critically ill COVID-19 patients admitted to the ICU of Hospital São José, a tertiary hospital in Lisbon, were included in this study. All infections were confirmed by real-time polymerase chain reaction (RT-PCR) testing. From these patients, 125 serum samples were collected between 12 November 2020 and 24 September 2021. Whenever samples were available, patients contributed more than one sample during the different stages of their ICU stay, but never more than one sample per temporal outcome group. Of the 125 samples, 52 correspond to patients who deceased in the ICU, while the remaining 73 samples are from patients who were ultimately discharged. Based on the time from sample acquisition to clinical outcome (discharge or death), all samples were categorized into three temporal groups: Group 1 (≤48 h, Early Outcome group), Group 2 (>48 h to ≤7 days, Intermediate Outcome group), and Group 3 (>7 days to ≤14 days, Late Outcome group). Vaccination status and viral strain differences were not considered in this study, as their distribution during the collection period was minimal and unlikely to influence the overall cytokine profile analysis. The full distribution of patients and samples per group is detailed in Table 1, Table 2 and Table 3. The current study was part of the Predictive Models of COVID-19 Outcomes for Higher-risk Patients Towards a Precision Medicine (PREMO) project and was approved by the hospital’s Ethics Committee. Informed consent was obtained from all patients or their immediate family members for data collection. All demographic, clinical, and laboratory data were extracted from the hospital’s electronic medical records system and subsequently anonymized.

### 2.3. Collection of Biological Samples

To mitigate ex vivo cytokine release triggered by coagulation-induced cell activation, samples obtained from peripheral blood were promptly processed immediately after visible clot formation. Blood was drawn into VACUETTE^®^ serum tubes (Greiner Bio-One GmbH, Kremsmünster, Austria) in accordance with standard phlebotomy protocols, without the use of anticoagulants. After collection during the hospital’s morning shift, blood samples were transported at 4 °C and kept at that temperature until serum isolation. Upon arrival at the processing laboratory, samples were centrifuged without delay at 3500 rpm for 10 min (Mikro 220T, Andreas Hettich GmbH & Co. KG, Tuttlingen, Germany). Serum was then aliquoted and stored at −80 °C until cytokine quantification.

### 2.4. Proteomic Cytokine Profile by Multiplex Immunoassay

Serum cytokine profiling of 21 proteins was performed using a targeted proteomic approach with the MILLIPLEX^®^ MAP Human High-sensitivity T Cell Panel (HSTC384-28K; Millipore, Merck, Germany) according to the manufacturer’s instructions: Interferon-inducible T cell alpha chemoattractant (ITAC), Granulocyte-Macrophage Colony-Stimulating Factor (GM-CSF), Fractalkine (also known as CX3CL1), IFN-γ, IL-10, macrophage inflammatory protein-3 alpha (MIP-3α, also known as CCL20), Interleukin-12p70 (IL-12p70), IL-13, IL-17A, IL-1β, IL-2, IL-21, IL-4, Interleukin-23 (IL-23), Interleukin-5 (IL-5), IL-6, Interleukin-7 (IL-7), IL-8, macrophage inflammatory protein-1 alpha (MIP-1α, also known as CCL3), macrophage inflammatory protein-1 beta (MIP-1β), and TNF-α. The assay was performed according to the manufacturer’s protocol, using a FLEXMAP^®^ 3D system with xPONENT^®^ software version 4.3 for data acquisition and analysis (Luminex Corporation, Austin, TX, USA).

### 2.5. Data and Statistical Analysis

The demographic variables analyzed in this study included age and gender [51,52], while clinical characteristics encompassed body mass index (BMI) [53,54]. Details regarding respiratory support modalities, specifically invasive mechanical ventilation (IMV) [55,56] and extracorporeal membrane oxygenation (ECMO) [57,58], were also recorded. These variables were selected due to their established prognostic relevance in COVID-19 and critically ill patients. Their distribution was compared between the two primary patient groups—those discharged and those deceased within the ICU—to minimize potential confounding effects. Statistical significance for these comparisons was defined as a two-tailed *p*-value less than 0.01.

Statistical analyses of demographic and clinical parameters were performed using Student’s t-test for normally distributed continuous variables and the Mann–Whitney U test for those not normally distributed. For categorical variables, chi-square (χ^2^) tests and Fisher’s exact tests were employed, with the latter reserved for small sample sizes. Continuous variables were summarized using medians and interquartile ranges (25th–75th percentiles), whereas categorical data were reported as absolute counts and percentages. All analyses were conducted using IBM SPSS Statistics software, version 27 (IBM Corp., Armonk, NY, USA), and GraphPad Prism, version 8.4.2 (GraphPad Software, San Diego, CA, USA).

Feature selection was performed using the fast correlation-based filter (FCBF) algorithm, which ranks features based on their ability to discriminate between discharged and deceased patient groups [59]. FCBF assigns a relevance score between 0 and 1 to each feature, with 1 indicating perfect discrimination and 0 indicating no discriminative power. Features scoring above 0 were retained for downstream modeling. Given the high dimensionality introduced by cytokine ratios, FCBF was chosen for its efficiency in eliminating redundancy, a task at which many alternative feature selection methods struggle in complex datasets. Feature selection was applied to the entire dataset prior to cross-validation, with the aim of identifying globally informative features rather than optimizing predictive performance for a specific data split [60]. Although this strategy carries a theoretical risk of data leakage, it ensures that the selected features capture the most robust biological signals. Future research with larger cohorts and independent training, validation, and test sets is warranted to further substantiate these findings [61].

To further explore patient group separations, the t-distributed stochastic neighbor embedding (t-SNE) method was applied. This unsupervised learning technique reduces high-dimensional data into two or three dimensions while maintaining local structure, facilitating visualization of clusters or group trends. Unlike traditional linear techniques such as Principal Component Analysis (PCA), t-SNE excels at preserving local distances between data points. This allows it to uncover subtle groupings and nonlinear relationships that PCA might miss, making it a particularly suitable choice for complex immunological datasets such as cytokine profiles [62]. In this study, t-SNE visualizations were generated using the top-ranked features identified by the FCBF algorithm (i.e., cytokines or cytokine ratios with the highest discriminative power between discharged and deceased patients). This ensured that the dimensionality reduction focused on the most informative variables, enhancing the separation of clinical outcomes in the visual space. Preprocessing steps included data normalization to reduce scaling bias, and the perplexity parameter was optimized to better preserve global structure, enhancing the visualization of broader patterns. Additionally, several supervised classification algorithms were developed, including k-nearest neighbors (kNN), naïve Bayes, random forest, support vector machine (SVM), and decision tree classifiers. Model training utilized a five-fold cross-validation strategy: the dataset was partitioned into five equal subsets, with iterative training on four subsets and testing on the fifth, ensuring every sample contributed to model validation once. This approach provided more reliable performance estimates and reduced overfitting risks by preventing the model from depending on any particular subset’s distribution.

For visualization of classifier performance, ROC curves were generated to display the true positive rate (sensitivity) against the false positive rate (1–specificity) for models trained on FCBF-ranked cytokine ratios for each group. Performance lines were plotted at a fixed threshold of 0.5, with equal false-positive and false-negative costs. The merge predictions from the folds option were applied to aggregate model outputs across cross-validation folds, providing a single consolidated ROC curve per group.

All data mining procedures, machine learning models, and performance visualizations were performed using the Orange: Data Mining Toolbox, version 3.36.2 (Bioinformatics Lab, University of Ljubljana, Ljubljana, Slovenia) [63].

Following feature selection, nomograms were constructed for each target class to facilitate intuitive interpretation of model outputs [64]. Nomograms graphically represent the impact of each selected feature on the outcome, leveraging the log odds ratio scale, which is widely accepted in clinical research. Features were ranked according to their FCBF-assigned importance, with graphical markers indicating the relative contribution of varying feature levels toward the outcome classification. Blue markers on the nomograms themselves serve as reference points for how the different ranges contribute to classification (e.g., discharged or deceased). Additionally, when the blue reference marker appears further to the right, it typically indicates a stronger contribution of that feature value to the predicted outcome, increasing the probability assigned to a specific target class. The top axis of the nomogram represents the effect size of each feature on an odds ratio scale, where values further to the right correspond to higher odds of classification into the positive class (e.g., deceased). The “total” axis below the predictors sums the individual contributions of each selected feature for a given patient sample, and this cumulative score is then mapped onto the final predicted probability using the probability scale at the bottom of the nomogram. This dual-axis layout allows for intuitive visualization of both the relative impact of each predictor and the overall predicted risk associated with a given cytokine ratio profile.

**Figure 2:**
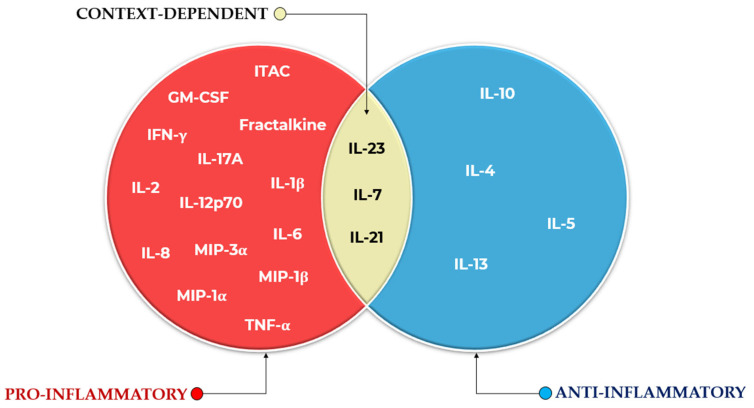
Categorization of the 21 cytokines analyzed in this study into pro-inflammatory (left, red), anti-inflammatory (right, blue), or context-dependent (center, yellow) groups.

To complement the cytokine assay, which measured 21 cytokines using the MILLIPLEX MAP 384-well Human High-sensitivity T Cell Panel, all possible biologically plausible cytokine ratio combinations were programmatically generated [65]. A maximum of three cytokines (summed) was allowed in both numerator and denominator. Pro-inflammatory cytokines (ITAC, GM-CSF, Fractalkine, IFN-γ, IL-17A, IL-1β, IL-2, IL-12p70, IL-23, IL-6, IL-8, MIP-1α, MIP-3α, MIP-1β, and TNF-α) were placed in the numerator, while anti-inflammatory cytokines (IL-10, IL-4, IL-13, IL-5, IL-7, and IL-21) were assigned to the denominator. It is acknowledged, however, that the functional classification of certain cytokines can vary depending on biological context. Another possible grouping of the 21 cytokines into pro-inflammatory, anti-inflammatory, or context-dependent categories, for the particular assay used in this study, is illustrated in Figure 2.

For instance, IL-5, IL-7, and IL-21 have been variably described as pro-inflammatory [66,67,68] or immunomodulatory/anti-inflammatory [69,70,71], depending on the context. Similarly, IL-2, though classically associated with pro-inflammatory T cell expansion [72], also plays a critical role in regulatory T cell maintenance and immune suppression [73]. Despite these nuances, a biologically pragmatic categorization was adopted here to streamline ratio generation. Following these assignments, the number of theoretical cytokine ratios was reduced from over 1.5 million to 19,229 biologically justified combinations. These, together with the 21 original cytokine concentrations (yielding 19,250 total features), were subjected to FCBF analysis to identify the strongest predictors of ICU mortality.

Lastly, cytokine distributions were individually assessed across discharged and deceased groups. Boxplots were generated to visualize data distribution, highlighting medians, interquartile ranges (IQR), and potential outliers (restricted to 1.5 × IQR to maintain scale clarity). The Shapiro–Wilk test was applied to evaluate normality within groups, guiding the use of Student’s *t*-test for normally distributed variables and the Mann–Whitney U test for non-normally distributed ones. Statistical significance was annotated directly on the plots alongside corresponding *p*-values.

## 3. Results and Discussion

### 3.1. Study Population Characteristics

The current study included 89 ICU patients (125 total samples) from a tertiary hospital in Lisbon, of whom 34 deceased in the ICU (corresponding to 52 samples), with the remaining 55 patients (corresponding to 73 samples) being eventually discharged from the ICU (and surviving, that is, even after being discharged to the infirmary, patients survived still). The 125 samples were then distributed over three distinct groups (Group 1: ≤48 h, Group 2: >48 h to ≤7 days, Group 3: >7 days to ≤14 days), where it was evaluated for the two patient target classes (discharged or deceased), if and where they differed statistically (*p* < 0.01) in terms of demographic characteristics (age, gender) or clinical variables (ECMO, IMV, BMI). This was carried out to gain a better understanding on the potential effects of confounding variables [65]. It was found that only for the variable age, there was a significant difference (*p* < 0.01) for both groups 1 and 2, which is easy to understand and recognized in the literature, in which older ICU patients are more vulnerable and prone to having complications that eventually lead to death. Table 1, Table 2 and Table 3 below reflect these findings for groups 1–3.

### 3.2. Commonly Reported Cytokines and Ratios

Cytokines, simple cytokine ratios, and traditional severity scores such as APACHE II, SAPS II, and SOFA have been widely investigated as prognostic tools in critically ill populations. However, their predictive utility often remains context-dependent, with most studies focusing on specific syndromes such as sepsis, ARDS, or COVID-19. In addition, due to the inherent complexity of ICU research, many studies necessarily involve relatively limited and specialized patient cohorts. In the current study, all patients were critically ill with confirmed COVID-19, providing a uniform population while avoiding heterogeneity between COVID-19 and non-COVID-19 cases. Although smaller cohort sizes are common in ICU research, efforts were made to minimize confounding effects by applying analytical strategies capable of extracting robust predictive features from available data. To provide a foundation for the analysis, the following section summarizes the behavior of commonly reported cytokines, ratios, and severity scores across the three time-based patient groups (Group 1: ≤48 h, Group 2: >48 h to ≤7 days, Group 3: >7 days to ≤14 days), as presented in Table 4.

Traditional severity scores such as APACHE II, SAPS II, and SOFA have been widely employed to predict outcomes in ICU settings. Their prognostic utility has been validated in a range of critical care scenarios, although performance can vary depending on patient cohorts and disease contexts. For instance, the SOFA score has shown a fair discriminatory ability for predicting ICU mortality in sepsis cohorts, achieving AUC values around 0.713, outperforming APACHE II in some studies [74].

Similarly, SAPS II has demonstrated moderate discrimination for mortality, with AUC values often ranging between 0.79 and 0.83 in general ICU cohorts [75,76], although performance varies by patient population and requires periodic recalibration. Meta-analyses indicate that APACHE II models generally perform comparably to or slightly better than SOFA at admission, with sequential SOFA measurements offering enhanced prognostic ability [77]. In COVID-19 cohorts specifically, APACHE II and SOFA scores have shown moderate predictive ability, with APACHE II achieving AUCs between 0.65 and 0.85 depending on the cohort [78,79].

Demichev et al. showed limited prognostic capability of traditional clinical scores (APACHE II, AUC = 0.68, *p* = 0.05; SOFA, AUC = 0.65, *p* = 0.11) in predicting COVID-19 ICU outcomes [80]. These results parallel our manuscript findings (Table 4), where APACHE II, SAPS II, and SOFA scores failed to reach statistical significance for mortality discrimination, underscoring the limitations of conventional scoring systems and advocating for enhanced biomarker-based predictive strategies.

Serial SOFA score trends (i.e., SOFA scores tracked over time) have demonstrated strong associations with 28-day mortality in ICU-admitted COVID-19 patients, particularly when assessing mean SOFA scores across the first 96 h [81]. For example, mean SOFA scores during the initial 96 h ICU stay showed AUC values around 0.77 for predicting 28-day mortality, outperforming initial SOFA scores alone [81]. In dynamic ICU mortality models for COVID-19, the integration of clinical severity scores alongside daily patient data has further enhanced predictive capabilities [82]. Despite moderate discriminatory ability overall, severity scores remain critical baseline tools in both COVID-19 and non-COVID-19 critical illness [83,84].

Cytokines have long been studied as complementary biomarkers to refine prognostic assessments in critical care; however, when used as standalone biomarkers, they face inherent limitations. IL-6, for example, has shown highly variable prognostic accuracy across studies and cohorts. In community-acquired pneumonia, IL-6 reached an AUC of 0.934 with high sensitivity and specificity (84% and 87%, respectively) [85]. However, other studies—such as in COVID-19—have reported far lower values, with AUCs around 0.531 and sensitivity as low as 15% [86].

In critically ill COVID-19 patients, using the plasma proteome of a cohort (Germany), by using 57 proteins, validated by an independent cohort (Austria), a machine learning model using SVM achieved high predictive power (AUC = 1.0, *p* < 0.001), being able to predict the outcome (survival or death). Though the study concludes that plasma proteomics can yield highly accurate prognostic predictors in intensive care, it does not address potential limitations regarding real-time clinical applicability, such as turnaround time, cost-effectiveness, or feasibility in routine settings [80]. Vanderkamp et al. highlighted high IL-6 concentrations as critical biomarkers associated with lethal COVID-19 [87]. This closely mirrors our findings, where IL-6 was significantly elevated in deceased patients, particularly within group 1 (Table 4 and Table 5). This agreement reinforces the prognostic relevance of IL-6 as a robust indicator of disease severity and mortality risk in COVID-19 ICU patients.

While IL-6 may outperform markers like C-reactive Protein (CRP) in some settings, its predictive metrics often hover around 0.70, emphasizing the need for more consistent and universally applicable biomarkers [88]. Notably, although CRP remains a well-established marker of inflammation, particularly in infectious settings, its prognostic use for ICU mortality was not prioritized in this study, which focused on systemic severity scores instead. Additionally, cytokine levels are often reported without considering their interaction with other inflammatory mediators, curbing their predictive power.

Recent studies, particularly in COVID-19 cohorts, have reinforced these findings by demonstrating that elevated levels of IL-10, CCL2, CXCL9, and CXCL10 (the latter three also referred to as MCP-1, MIG, and IP-10, respectively) are associated with increased mortality among patients with severe COVID-19 in the ICU [89]. Furthermore, IL-1β and IL-10 imbalances were also linked to worse outcomes in critically ill COVID-19 patients, with lower IL-1β and higher IL-10 levels predicting mortality at day 7 [90].

Huang et al. also observed that cytokines such as IL-10 and IL-6 were significantly elevated during progression from mild to severe COVID-19, emphasizing their role in disease exacerbation [91]. Similarly to our findings, IL-10 showed elevated levels in deceased ICU patients within early and intermediate groups, reaching statistical significance in group 2 (Table 4 and Table 5). However, in the current study, IL-10 was paradoxically higher in discharged patients within the late group, highlighting its dual role in immune regulation and suggesting its temporal context-dependent prognostic value.

In sepsis studies, dynamic declines in IL-6 and TNF-α levels after ICU admission correlated with survival, whereas persistently elevated levels were associated with non-survival [92]. Adding nuance, another study showed that IL-6 levels were predictive of mortality predominantly in younger sepsis patients, with less clear associations in elderly populations [93]. Other cytokines, such as IFN-γ, IL-17A, GM-CSF, IL-1β, and fractalkine, have also demonstrated associations with ICU mortality, although their clinical application remains under active investigation [94,95,96,97,98,99,100,101,102]. More specialized studies have explored chemokine profiles, identifying markers like MCP-3 and IL-8 as potent discriminators of ICU mortality in COVID-19. In a cohort of 242 critically ill patients, a scoring algorithm based on these cytokines achieved an AUC of 0.985 in the internal test set and 0.969 in the external validation set, highlighting its strong predictive performance [103]. Importantly, in elderly critically ill COVID-19 patients with renal failure, mortality was shown to be associated with disease severity rather than cytokine absorption interventions, underscoring the complexity of interpreting cytokine data [104].

While individual cytokines provide valuable information, cytokine ratios may better reflect immune dysregulation and improve prognostic accuracy. Ratios such as IL-6/IL-10 and TNF-α/IL-10 have been proposed as superior markers compared to single cytokines alone [94,95,96].

Lu et al. discussed the suboptimal efficacy of IL-1 and IL-6 blockade in preventing COVID-19 mortality, suggesting complexity in cytokine interplay [105]. Our results similarly highlighted IL-1 and IL-6 elevations predominantly in deceased patients in early and intermediate groups, while observing IL-1 elevation in discharged patients in the late group (Table 4), reflecting dynamic inflammatory shifts. Notably, our IL-6/IL-10 ratio emerged consistently elevated exclusively in deceased groups (see Table 4), emphasizing the critical balance between pro- and anti-inflammatory responses as a precise prognostic indicator in severe COVID-19. Indeed, studies have demonstrated that a low IL-1β/IL-10 ratio was associated with increased mortality risk in severe COVID-19 ICU patients, particularly at day 7 of ICU stay [90]. Similarly, excessive concurrent elevations of IL-6 and IL-10 have been linked to poor outcomes in severe COVID-19 patients [96]. Recent studies have also highlighted the relevance of immune cell-normalized cytokine ratios. For instance, an elevated IL-6/lymphocyte ratio was associated with increased mortality risk in severe pneumonia patients requiring ICU admission [106], whereas a high IL-10/lymphocyte ratio was predictive of 28-day mortality in patients with severe sepsis [107]. Additionally, the neutrophil/lymphocyte ratio (NLR) has been identified as a prognostic marker for ICU admission risk among COVID-19 patients, particularly in diabetic populations [108]. Moreover, analyses have shown that MCP-3/IL-8 scoring systems can accurately predict ICU mortality and cytokine storm severity in COVID-19 patients, offering clinical utility for risk stratification [103]. General associations between IL-8 elevations and organ failure were also observed, supporting the rationale for integrating cytokine ratios into clinical decision-making [109].

Despite cytokine ratios offering improvements over isolated markers, increasing evidence suggests that multi-cytokine signatures and integrated biomarker panels provide even greater prognostic power. Research increasingly points toward comprehensive cytokine signatures or multivariate biomarker panels as more robust predictors of clinical outcomes. Persistently high levels of IL-6 and IL-10, and elevated IFN-γ and IL-17A, have been associated with multi-organ failure and ICU mortality [97,98,99]. These trends underscore the promise of cytokine-based prognostication, especially when leveraged alongside computational tools such as machine learning, which allow for high-dimensional biomarker pattern recognition [50,110,111].

A key practical consideration in cytokine biomarker research is the selection of an appropriate biofluid. Serum is commonly chosen for its stability, reproducibility, and suitability for extended storage. Derived from whole blood, serum provides a standardized matrix for exploring systemic immune responses [112,113]. Its applicability has been demonstrated across various studies examining inflammation, sepsis, cancer, and critical illness [114,115,116,117]. Building on these considerations, the following section examines how the behavior of severity scores, cytokines, and cytokine ratios in our cohort converges with or diverges from existing literature findings.

#### Convergence with and Divergence from the Literature

In the present study, the analysis of severity scores, cytokine levels, and simple cytokine ratios revealed varying degrees of discriminatory capacity between deceased and discharged ICU COVID-19 patients, stratified across three time-based groups according to sample collection timing.

In Group 1 (≤48 h), significant differences were identified for two individual cytokines: IL-6 (*p* = 0.002) and IL-8 (*p* = 0.002), both elevated in deceased patients, achieving significance at the 1% level (Table 4). Among simple ratios, the IL-6/IL-10 ratio (*p* = 0.002) also reached strong statistical significance (Table 4). Additionally, other ratios, including IL-6/lymphocytes (*p* = 0.010), IL-10/lymphocytes (*p* = 0.040), neutrophil/lymphocyte ratio (*p* = 0.015), and TNF-α/IL-10 ratio (*p* = 0.048), were significant at the 5% level (Table 4). Traditional severity markers (APACHE II, SAPS II, SOFA) did not distinguish between outcomes in this group (Table 4). These findings reinforce the emerging evidence linking early elevations of IL-6 and IL-8 to poor outcomes in critical COVID-19 cases [30,31,89,90].

For Group 2 (>48 h to ≤7 days), a broader set of significant differences was observed. IL-8 (*p* = 0.001) and Fractalkine (*p* = 0.003) levels were significantly higher in deceased patients at the 1% level, while IL-10 (*p* = 0.015) was also elevated, achieving significance at the 5% threshold (Table 4). Regarding simple ratios, IL-6/lymphocytes (*p* = 0.001), IL-10/lymphocytes (*p* < 0.001), and neutrophil/lymphocyte ratio (*p* = 0.009) were significantly higher among deceased patients, all at the 1% level. The TNF-α/IL-10 ratio (*p* = 0.018) also showed significance at the 5% level (Table 4). As in Group 1, traditional severity scores failed to demonstrate significant discrimination. These results align with previous literature highlighting the prognostic relevance of IL-8 elevations, heightened IL-10 responses, and altered neutrophil/lymphocyte ratios in critically ill populations, including COVID-19 patients [89,90,106,107].

In Group 3 (>7 days to ≤14 days), fewer statistically significant differences were identified. Only IFN-γ (*p* = 0.001) distinguished between groups, with higher levels found in discharged patients (Table 4). Among simple ratios, the IL-6/IL-10 ratio (*p* = 0.029) reached significance at the 5% level, favoring higher values in deceased patients (Table 4). No severity scores demonstrated discriminatory ability at this stage (Table 4). The relative lack of significant differences in this group is consistent with existing knowledge that cytokine-driven pathophysiological events, including the cytokine storm and associated immune dysregulation, are most pronounced during the early phase of critical illness, typically within the first 48 h to the first week—a timeline not characteristic of this specific group. By the second week, immune responses tend to stabilize or diversify depending on individual trajectories and therapeutic interventions, reducing the likelihood of detecting sharp biomarker differences [118,119]. The specific increase in IFN-γ among discharged in this later group aligns with previous findings indicating a significant decline in IFN-γ over the first week of ICU stay among the deceased group, reinforcing its potential role as a favorable prognostic indicator at extended time points [90]. Similarly, while TNF-α is often associated with poor outcomes in early-phase sepsis, the observed elevation in the discharged here is consistent with prior findings in acute renal failure, where TNF-α did not predict mortality and was instead higher among discharged, suggesting a more complex or time-dependent role for this cytokine in critically ill patients [26]. It should also be noted that the high percentage of missing data for lymphocyte-based ratios—ranging from 30% to 57.9% depending on the group and outcome—could have contributed to underestimating significant findings in these comparisons (Table 4).

Overall, these results converge with prior studies emphasizing the predictive value of early pro-inflammatory and anti-inflammatory cytokine imbalances in COVID-19 ICU mortality. The persistent elevations of IL-6 and IL-8 in earlier groups, alongside the emerging importance of immune cell-adjusted ratios, reinforce the potential clinical value of integrated cytokine profiling. Divergences at later stages likely reflect a shift from acute inflammatory phenomena to more heterogeneous chronic processes or variations in treatment strategies.

Building on these findings, the next section will explore the univariate behavior of the full cytokine panel, presenting the distribution of all 21 cytokines across outcome groups, supported by visualizations of statistically significant differences and comparative metrics including absolute differences, percentage differences, and fold changes (FC).

### 3.3. Univariate Cytokine Analysis

This section presents the median and IQR values for all 21 cytokines across the ICU patients (discharged versus deceased), stratified by the three time-based groups (Group 1: ≤48 h, Group 2: >48 h to ≤7 days, Group 3: >7 days to ≤14 days). To characterize differences between outcome groups, additional metrics were computed, including the absolute difference in medians, percentage difference (% diff), and fold changes. In this analysis, deceased patients were used as the reference group for all comparisons. The absolute difference captures the magnitude of change, while the percentage difference conveys directionality—indicating whether cytokine concentrations are relatively elevated or reduced in discharged patients compared to the deceased. Fold changes further highlight relative up- or down-regulation of cytokines across groups.

**Figure 3:**
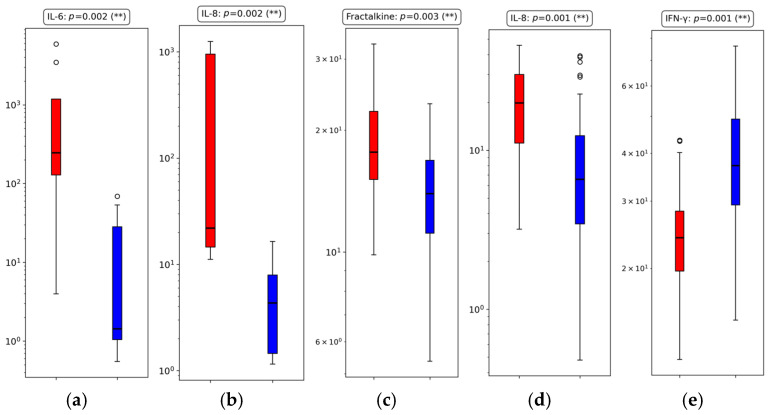
Boxplots displaying cytokine levels with statistically significant differences between deceased (red) and discharged (blue) ICU COVID-19 patients across the three time-based groups. Panels (**a**,**b**) correspond to Group 1 (≤48 h), panels (**c**,**d**) to Group 2 (>48 h to ≤7 days), and panel (**e**) to Group 3 (>7 days to ≤14 days). **: *p* < 0.01.

The most significant cytokine differences (*p* ≤ 0.01) identified across the three time-based groups are summarized in Figure 3, while all cytokines, including those that did not reach statistical significance (*p* > 0.01), are listed in Table 5. For each group, Figure 3 displays boxplots for cytokines that were significantly different between outcome groups, offering a visual comparison of their distributions. For the complete set of 21 cytokines, the corresponding median and IQR values, absolute differences, percentage differences, and fold changes are detailed in Table 6 for the three time-based groups.

#### 3.3.1. Group 1: Early Outcome (≤48 h)

In the early mortality group, IL-6 (*p* = 0.002) and IL-8 (*p* = 0.002) were the only cytokines to reach statistical significance at the 1% level. Both were elevated in deceased patients, reflecting severe inflammatory processes or late-stage exacerbations, rather than the initial cytokine storm and acute inflammation typically observed during the early phase of critical COVID-19 shortly after ICU admission [30,31,89,90]. IL-6 demonstrated a particularly striking FC of 244.56, with a 99.6% increase compared to discharged patients. IL-8 also showed a substantial 70.7% increase, with an FC of 3.41, reinforcing its strong pro-inflammatory signal early in the disease course.

Regarding MIP-3α, while the fold change is relatively modest (1.27), its directional trend aligns with reports of MIP-3α contributing to leukocyte recruitment in severe disease [120,121], indicating that this cytokine may still hold clinical relevance when considered as part of broader multi-cytokine panels or integrated predictive models. Among the cytokines previously highlighted in Table 4, IL-10, despite a non-significant *p*-value (*p* = 0.173), exhibited a near-doubling in median values (41.77 vs. 21.54 pg/mL), with a 48.4% increase and a 1.94 FC in deceased patients. Although underpowered in this group, this trend is biologically coherent, reflecting a compensatory anti-inflammatory response. TNF-α (*p* = 0.066) followed a similar pattern, with a 42.8% elevation and a 1.75 FC change, further supporting its early relevance despite borderline significance. Conversely, IFN-γ, IL-1β, and GM-CSF remained stable between groups, showing less than 10% difference and fold changes close to 1.0.

Altogether, these findings underscore IL-6 and IL-8 as robust early biomarkers of mortality, with additional support for IL-10, TNF-α, and MIP-3α as potentially informative targets that may reach significance in larger cohorts or multivariate models. Their directional shifts, despite varying significance levels, reinforce the early inflammatory burden in deceased patients and highlight candidates for early risk stratification.

#### 3.3.2. Group 2: Intermediate Outcome (>48 h to ≤7 Days)

In the intermediate mortality group, IL-8 (*p* = 0.001) and Fractalkine (*p* = 0.003) were significantly elevated in deceased patients at the 1% level. IL-8 demonstrated a strong 70.13% increase and a 3.35-fold change, confirming its sustained relevance as a pro-inflammatory marker beyond the initial 48 h of ICU care. Fractalkine, while more modest in effect, still showed a 21.19% increase and a 1.27 FC, pointing to its possible involvement in prolonged immune activation and vascular signaling [122,123,124].

At the 5% level, IL-10 (*p* = 0.015) was higher in deceased patients, with a 34.02% increase and a 1.52 FC, suggesting the persistence of counter-regulatory anti-inflammatory mechanisms. Additional cytokines reaching statistical significance at this level included IL-7 (*p* = 0.033), IL-23 (*p* = 0.020), and MIP-1α (*p* = 0.034). IL-7 showed a 23.86% increase and a 1.31 FC in deceased patients, while IL-23 exhibited a 29% increase and a 1.41 FC, both indicating a potential role in immune regulation and inflammatory signaling during this intermediate stage. MIP-1α showed a 20.48% increase and a 1.26 FC, potentially reflecting chemotactic activity (i.e., directed immune cell migration) contributing to immune cell recruitment. Although none of these cytokines displayed fold changes as large as IL-6 or IL-8, their statistical relevance suggests possible biological involvement and supports their inclusion in future exploratory models.

From the set of cytokines also discussed in Table 4, TNF-α did not reach significance (*p* = 0.102) and showed only a 7.07% increase with a 1.08-fold change, indicating limited discriminatory value at this intermediate stage. In contrast, IL-6, though also non-significant (*p* = 0.079), presented a notable 81.36% increase and a 5.36-fold change, suggesting continued systemic inflammation that may be biologically relevant despite the lack of statistical power.

Taken together, these findings highlight IL-8 and Fractalkine as key discriminators in the intermediate phase, with additional significant contributions from IL-7, IL-10, IL-23, and MIP-1β, supported by directional changes in IL-6. Their combined behavior underscores a transition period where both inflammatory and anti-inflammatory forces remain active and contribute to outcome divergence.

#### 3.3.3. Group 3: Late Outcome (>7 Days to ≤14 Days)

In the late mortality group, IFN-γ was the only cytokine to reach statistical significance (*p* = 0.001). It was markedly higher in discharged patients, with a 51.72% decrease in deceased individuals and a fold change of 0.66, reinforcing its potential role as a favorable immune mediator in later stages of recovery. These findings are consistent with prior observations of declining IFN-γ levels in patients who ultimately do not survive prolonged ICU stays [90]. No other cytokines were statistically significant at either the 1% or 5% level. However, several demonstrated biologically relevant directional trends. IL-6, though not statistically significant (*p* = 0.186), showed a substantial 79.97% increase and a 4.99-fold change in deceased patients, suggesting ongoing pro-inflammatory activity in late-stage critical illness. IL-8 followed a similar pattern, with a 40.62% increase and a 1.68-fold change, despite a *p*-value of 0.070.

Among the cytokines tracked in Table 4, TNF-α and IL-10 both showed slightly higher values in discharged patients, with negative percentage differences (−7.31% and −7.76%, respectively) and fold changes of 0.93. While not statistically significant, these inversions from earlier patterns may suggest shifting regulatory dynamics or immune exhaustion in deceased patients. IL-1β (−11.75%, FC = 0.89) and IL-23 (−10.00%, FC = 0.91) also trended higher in discharged patients but without meaningful statistical support.

Taken together, these results highlight IFN-γ as the most reliable marker of survival beyond the first week of ICU care. While the broader cytokine profile becomes less discriminatory at later stages, persistently elevated IL-6 and IL-8 in deceased patients may reflect residual inflammatory stress, albeit with reduced predictive value. Although cytokine monitoring has shown potential for continuous patient tracking and therapeutic guidance in certain inflammatory conditions [125], the prognostic utility of circulating cytokine measurements tends to diminish over time, likely due to their short half-lives and the increasing heterogeneity of immune responses in later stages of critical illness [126]. This limitation has led to the exploration of exosome-associated cytokines as potentially more stable biomarkers, although evidence for their prognostic value remains preliminary and inconsistent [127].

**Figure 4:**
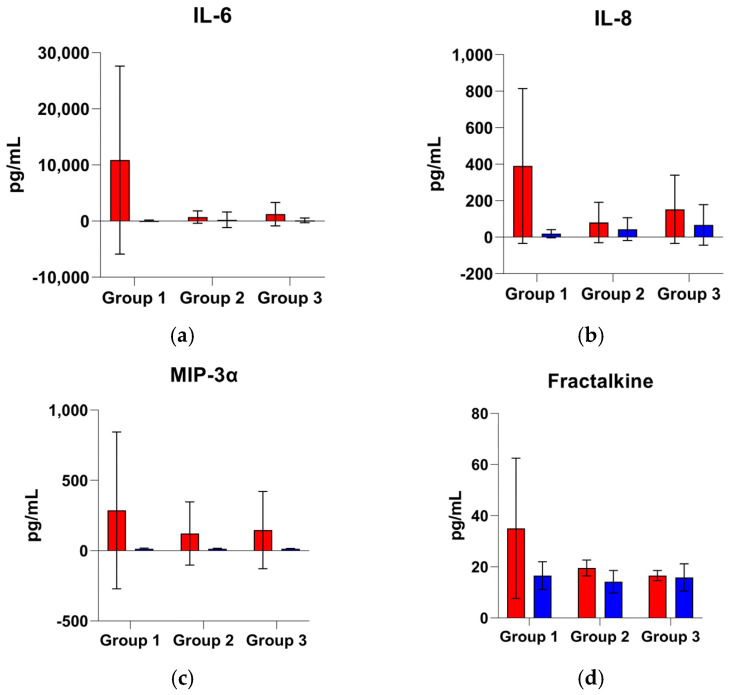

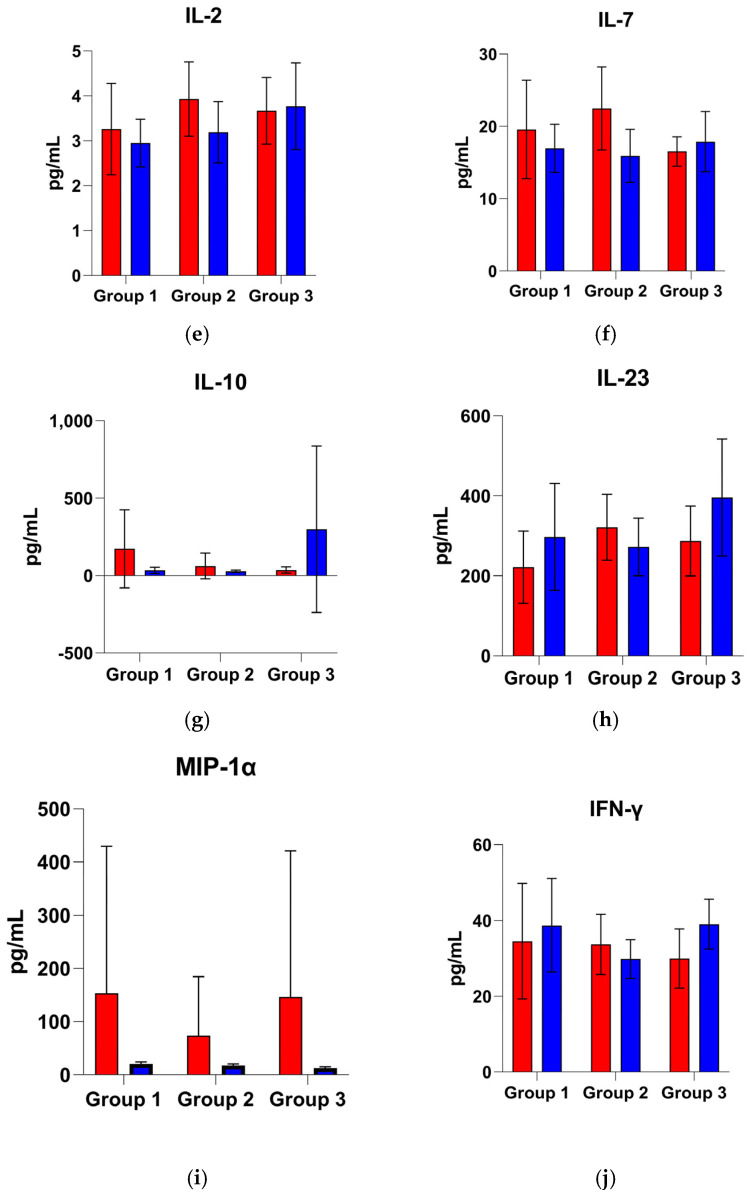
Bar charts representing average cytokine levels with 95% confidence intervals across three temporal groups (Group 1: ≤48 h; Group 2: >48 h–7d; Group 3: >7d–14d), stratified by patient outcome. Red bars represent deceased and blue bars represent discharged ICU COVID-19 patients. Panels (**a**–**c**) correspond to Group 1 cytokines identified as statistically significant in Table 5; panels (**d**–**i**) correspond to Group 2 cytokines (with panel b/IL-8 repeating), and panel (**j**) represents the sole significant cytokine identified for Group 3.

To illustrate the magnitude and progression of cytokine differences identified as statistically significant (Table 5) in the univariate analysis, Figure 4 displays bar charts representing mean cytokine levels (Fractalkine, IFN-γ, IL-2, IL-6, IL-7, IL-8, IL-10, IL-23, MIP-3α, and MIP-1α) with 95% confidence intervals across three time-based groups stratified by patient outcome (deceased and discharged). A linear scale was used to enable transparent comparison of cytokine levels between groups, though it may visually compress smaller fluctuations. This representation enhances the visual interpretation of outcome-dependent cytokine trajectories and highlights both marked and subtle trends over time.

Several pro-inflammatory cytokines (IL-6, IL-8, MIP-3α) exhibit an initial decline from the acute phase followed by a late uptick in the deceased group, contrasting with more sustained decreases in the discharged group. For example, IL-6 and IL-8, key mediators of systemic inflammation, drop in the ≤48 h window for both outcomes, but by the second week (Group 3), levels in deceased patients rebound markedly, whereas those in discharged patients remain relatively low. The consistent re-elevation of IL-6 and IL-8 in those who succumb is especially striking given their known linkage with persistent inflammation and organ failure. This pattern suggests a failure of immune resolution or a second inflammatory surge in patients with poor outcomes, potentially driven by unresolved infection, secondary insults, or patient factors like advanced age and comorbidities that predispose to prolonged systemic inflammation. These findings align with reports that persistent high IL-6 and IL-8 levels often precede death in sepsis, underscoring that an early tapering of pro-inflammatory signals, without late rebounds, is a hallmark of recovery [128]. In short, the deceased in our cohort remained “stuck” in an inflammatory state or even experienced a late cytokine rebound, whereas the discharged showed a more continuous damping of these responses over the two-week period, indicating successful inflammatory resolution. MIP-3α shows a similar trend with an early fall though not as pronounced as the two before, with a resurgence in fatal cases > 7 days post-onset. Fractalkine, a monocyte-attractant chemokine, displays a slightly different behavior, with a downward trend in both the deceased group and discharged group, with levels of this last one slightly elevated in the second week of ICU stay.

In contrast, another subset of cytokines demonstrates crossover trends in which levels in the discharged eventually exceed those in the deceased by the late phase. IL-2, IL-7, IL-10, and IL-23 exemplify this behavior. Early on (≤48 h and up to 7 d), these mediators are still higher in the deceased group, but by >7–14 d (Group 3) the discharged patients have higher average concentrations, even though globally inferior to that of groups 1 and 2 (except for IL-23 discharged in group 3 that displays the highest average values of the cytokine of any group for both deceased and discharged target). This late predominance in the discharged group suggests an association with protective or recovery-driven immune activity. IL-2 and IL-7 are crucial for lymphocyte proliferation and survival; their relative increase in discharge by week 2 may reflect the restoration of adaptive immunity as the hyperacute inflammation subsides. In particular, IL-7 is known to reverse sepsis-induced lymphopenia and improve host defense in experimental models, supporting the idea that rebounding lymphocyte-supportive cytokines benefit recovery [129]. IL-23, which drives Th17 responses, also becomes higher in late survivor groups, hinting that a controlled Th17 immune activation might aid in clearing persistent infection or healing tissues once the initial cytokine storm is tempered.

Most notably, IL-10 (an anti-inflammatory cytokine) is significantly elevated in discharge during the >7 d period, surpassing the levels in those who died by quite a margin. This suggests that recovering patients mount a stronger compensatory anti-inflammatory response in the subacute phase, which could help shut down excessive inflammation and promote tissue recovery. IL-10 is well recognized for its immunoregulatory, organ-protective effects in critical illness: for example, IL-10 deficiency in septic models leads to uncontrolled inflammation and early death, whereas IL-10 administration improves survival [130]. Thus, the higher late IL-10 in discharged patients aligns with its protective role in resolving inflammation. It is interesting to note that while IL-10 is often found to spike alongside pro-inflammatory cytokines in the initial cytokine storm (and can even be markedly elevated in early deceased patients [128] as a counter-regulatory response), our data indicate that a sustained or secondary rise in IL-10 is characteristic of survivors (i.e., discharged), not the terminally ill. This timing nuance implies that an adequate, well-timed anti-inflammatory response (as evidenced by IL-10 and possibly IL-7/IL-2 surges) is a possible marker of successful recovery, whereas patients who fail to mount or maintain this late anti-inflammatory phase may remain in an unchecked inflammatory state and succumb. Overall, the crossover of IL-2, IL-7, IL-23, and IL-10, with the discharged group ultimately exhibiting higher levels, points to these factors as potential markers of immune recovery and resilience, helping tilt the balance from destructive inflammation toward healing and homeostasis in the later stage of critical illness.

Finally, IFN-γ and MIP-1α showed distinct and somewhat atypical patterns. IFN-γ (a classic Th1 cytokine important for macrophage activation and cellular immunity) deviated from the classic “pro-inflammatory = worse outcome” paradigm by being consistently higher in discharged patients at multiple time points (groups 1 and 3). Rather than indicating harm, an elevated IFN-γ in discharge likely reflects a more effective cell-mediated immune response that aids in pathogen clearance and immune regulation. In severe sepsis, an inability to produce IFN-γ (often due to immune exhaustion or paralysis) is associated with poor outcomes, whereas restoring IFN-γ activity can be beneficial. Indeed, clinical studies have shown that therapeutic IFN-γ can reverse sepsis-induced immunosuppression, restoring immune function and facilitating the clearance of infections [131]. Thus, the higher IFN-γ observed in discharge here may denote an intact or reactivated cellular immune competence. Essentially, these patients avoid the detrimental “immunoparalysis” phase and retain the capacity to fight off infections. This finding underscores IFN-γ’s dualistic nature: while pro-inflammatory, it also orchestrates critical host defenses, and in context it appears more of a hallmark of effective immunity than of injurious inflammation.

On the other hand, MIP-1α, a chemokine that recruits neutrophils and macrophages, displayed high variability and only subtle average differences between outcomes. Patients in the deceased group tended to have higher MIP-1α levels by the second week, but the very wide confidence intervals indicate considerable patient-to-patient heterogeneity. Some individuals (including certain fatal cases) may have experienced massive late surges in MIP-1α, perhaps in the setting of secondary infection or an overwhelming late inflammatory event, while others had minimal changes, resulting in large error bars. This variability suggests that MIP-1α is not a consistently reliable marker of outcome in our cohort. In fact, prior work has noted that MIP-1α levels do not always correlate with sepsis severity or mortality [132], likely because its release can be influenced by where the infection is located or by genetic factors, leading to an inconsistent pattern. The lack of a clear trend for MIP-1α in our data might indicate that the inflammatory response is highly heterogeneous in critically ill patients with some following the representative trajectories of cytokines like IL-6/IL-10, while others have atypical surges in chemokines like MIP-1α. From a clinical standpoint, the large confidence interval for MIP-1α warns that any single-point measurement of this chemokine could be misleading. Only extreme elevations, such as those seen in some deceased patients, are likely to signal a particularly aggressive inflammatory state or impending deterioration, while moderate changes remain difficult to interpret due to overlapping variability.

In summary, these cytokine profiles illustrate two distinct immunological trajectories in critical illness. One is characterized by ongoing or repeated pro-inflammatory activity (involving IL-6, IL-8, MIP-3α, and Fractalkine) without sufficient anti-inflammatory compensation, a pattern associated with mortality. The other shows a steady reduction in inflammation, along with a delayed increase in regulatory and immune-supporting cytokines such as IL-10, IL-7, IL-2, and IFN-γ, which is more often seen in patients who recover. These findings are biologically plausible and support the idea that patients who cannot resolve their initial cytokine storm or shift into a recovery phase are more likely to have poor outcomes. In contrast, those who manage to reach a more balanced immune state, marked by increased anti-inflammatory signaling and restoration of lymphocyte activity, tend to recover more successfully.

This understanding of how cytokine levels change over time could have important clinical applications. For example, persistent elevation or a second increase in IL-6 or IL-8 may indicate the need for more aggressive treatment or point to ongoing infection, while rising IL-10 and IFN-γ levels during the second week may signal a positive turn in immune function. In addition, the fact that IL-2 and IL-7 levels in discharged patients appear to catch up or slightly surpass those of deceased patients by Group 3 suggests that stimulating these cytokines later in the ICU stay may support immune recovery, particularly for those who do not mount this response naturally. By examining these trends alongside published evidence, such as the protective role of IL-10 in sepsis, the ability of IFN-γ to counteract immunosuppression, or the variable behavior of chemokines like MIP-1α, it is possible to gain a better understanding of how immune responses differ between discharged and deceased. This clearer picture may help guide future efforts to design immune-focused interventions for severe infections and improve patient outcomes.

The next section explores multivariate cytokine patterns, aiming to assess their combined discriminatory power across time-based groups using machine learning approaches.

### 3.4. Multivariate Cytokine Analysis

Despite the increasing interest in cytokine profiling for diagnostic purposes, including in the context of COVID-19 [126,133,134,135], the current literature continues to emphasize traditional laboratory markers, while multivariate and machine learning frameworks remain comparatively underexplored. This section investigates the potential of cytokine-based profiling for ICU mortality discrimination (deceased vs. discharged) across three time-based groups (Groups 1–3), defined by the interval from sampling to ICU discharge or death. The analysis incorporates supervised machine learning models and advanced feature selection techniques to evaluate both individual cytokines and programmatically generated cytokine ratios. Section 3.4.1 focuses on performance metrics derived from the original 21 cytokines, while Section 3.4.2 expands the analysis to include a total of 19,250 features—encompassing the 21 cytokines and 19,229 biologically plausible complex ratios derived from the original 21 cytokines. The findings underscore the added value of feature selection and the inclusion of complex ratios in enhancing classification performance and clustering structure.

#### 3.4.1. Individual Cytokine Analysis

The discriminative power of 21 individual cytokines for predicting ICU mortality was assessed across three distinct time-based patient groups, employing supervised machine learning models (kNN, Naïve Bayes, Random Forest, SVM, and Decision Tree). Models were evaluated based on sensitivity first, then specificity, and finally AUC. The detailed performance metrics are presented in Table 7.

Overall, the application of FCBF feature ranking enhanced the performance metrics of some models by reducing noise and allowing the models to focus exclusively on the most informative cytokines. Nonetheless, the improvements following feature ranking were inconsistent and modest across groups and models, highlighting important trade-offs between sensitivity, specificity, and AUC. Notably, the sensitivity-specificity trade-off underscores the limitations of interpreting AUC in isolation, consistent with cautions raised previously regarding the potential misleading nature of relying solely on AUC metrics [136] and reinforcing the recommendation to balance multiple performance metrics [137].

In Group 1 (≤48 h; *n* = 10 deceased, 10 discharged), the best-balanced performance post-FCBF ranking was achieved by the Decision Tree model, demonstrating a sensitivity of 0.700 and specificity of 0.600 (AUC = 0.575). Conversely, the SVM exhibited a notably higher AUC (0.850) but an unacceptable sensitivity of only 0.300, rendering it unsuitable for reliable mortality prediction. Importantly, significant age differences (*p* < 0.001) between deceased (median age = 68 years) and discharged (median age = 51 years) groups suggest potential confounding, as cytokine levels may correlate strongly with age-related inflammatory responses. The top-ranked cytokines identified in this early time window were IFN-γ, IL-6, and IL-23, known markers of acute inflammation and immune activation. IFN-γ and IL-6 have been previously highlighted as reliable mortality indicators in COVID-19 patients [138], while IL-23 has also been strongly associated with disease severity and in-hospital mortality [139]. Although FCBF substantially reduced the feature set to these three cytokines, improving interpretability and feasibility, overall sensitivity remained limited for critical clinical applications.

Group 2 (>48 h to ≤7 days; *n* = 23 deceased, 40 discharged) exhibited clear class imbalance, complicating the classification task. Post-ranking, Naïve Bayes yielded the highest AUC (0.781) but moderate sensitivity (0.435), while the Decision Tree offered the highest sensitivity (0.522), accompanied by moderate specificity (0.775) and lower AUC (0.613). The SVM again showed high specificity (0.925), most likely due to the disproportionate number of discharged samples versus deceased, but disappointingly low sensitivity (0.217), reinforcing concerns regarding sensitivity–specificity trade-offs. The two cytokines highlighted by FCBF in this intermediate time frame, IL-7 and IL-8, reflect different aspects of immune regulation. IL-7 is associated with lymphocyte survival and proliferation and has been validated as a predictive cytokine for COVID-19 outcomes in longitudinal measurements [140]. IL-8, involved in neutrophil activation and recruitment, has consistently been shown to correlate with COVID-19 severity and prognosis, demonstrating strong predictive capacity for disease outcomes [141,142]. These cytokines’ prominence suggests ongoing complex immune dynamics beyond the acute initial inflammatory phase, highlighting the difficulty of achieving satisfactory predictive performance using single cytokines in a moderately imbalanced dataset and underscoring the need for complementary analytical approaches.

In Group 3 (>7 days to ≤14 days; *n* = 19 deceased, 23 discharged), classification performance post-FCBF ranking was generally poor. The Decision Tree provided the highest sensitivity (0.684) but at the cost of low specificity (0.348) and marginal AUC (0.521). SVM, despite exceptional specificity (0.957), failed entirely to identify deceased patients (sensitivity = 0.000), illustrating critical limitations. Only IL-8 was retained following FCBF selection, reflecting its persistent role in prolonged inflammatory responses, though evidently insufficient, when used on its own, to achieve a robust predictive capability. The reduction to a single cytokine prevented effective multivariate analysis and indicated a diminishing cytokine signature in predicting mortality during prolonged ICU stays. This aligns with the known patterns of cytokine storms and immune response divergence predominantly occurring early in the disease progression, diminishing over extended critical illness durations [119].

**Figure 5:**
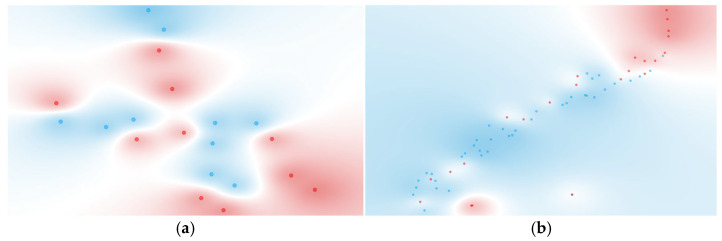
t-SNE visualization of Fast Correlation-Based Feature-ranked cytokines used for Naïve Bayes classification of mortality in ICU COVID-19 patients (deceased: red, discharged: blue). Visualization for Group 1 (≤48 h) using 3 ranked cytokines (**a**), and for Group 2 (>48 h to ≤7 days) using 2 ranked cytokines (**b**). Group 3 is not represented, as t-SNE requires a minimum of two features. Feature selection was performed using the Fast Correlation-Based Filter method.

Visualization via t-SNE (Figure 5) reinforces these quantitative results. Group 1 showed moderate clustering between deceased and discharged patients, indicative of some underlying structure captured by the top-ranked cytokines. Group 2 displayed weaker, yet discernible separation, corresponding to a modest predictive performance. Group 3 could not be visualized due to the reduction to a single cytokine post-FCBF, underscoring the practical constraints of aggressive feature selection in datasets with inherently limited discriminatory power.

In summary, the models trained on individual cytokines, while benefiting from FCBF feature reduction in interpretability and implementation feasibility, did not achieve clinically meaningful predictive accuracy across the three time-based groups. Limitations such as small sample sizes, class imbalance (notably in Group 2), and potential confounding by age were evident and likely impacted model performance. Consequently, reliance on single cytokine concentrations alone appears insufficient for robust ICU mortality prediction in COVID-19 patients. Therefore, Section 3.4.2 explores the integration of programmatically generated cytokine ratios, aiming to capture more nuanced immunological relationships and potentially enhance predictive performance.

#### 3.4.2. Computationally Generated Cytokine Ratios

**Figure 6:**
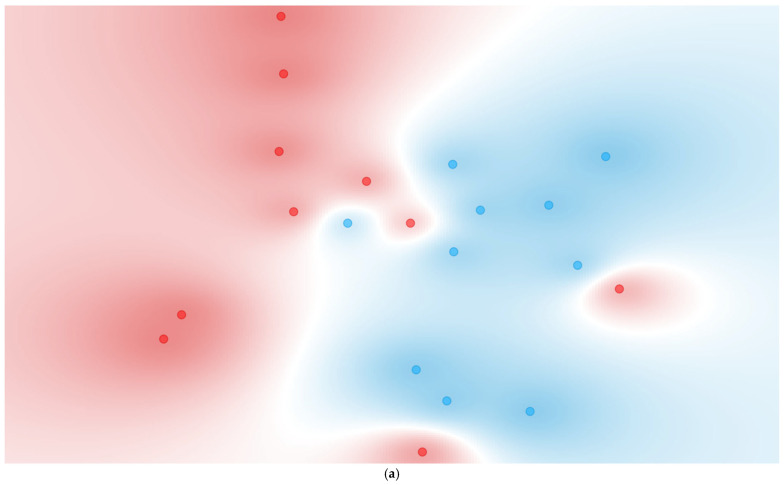

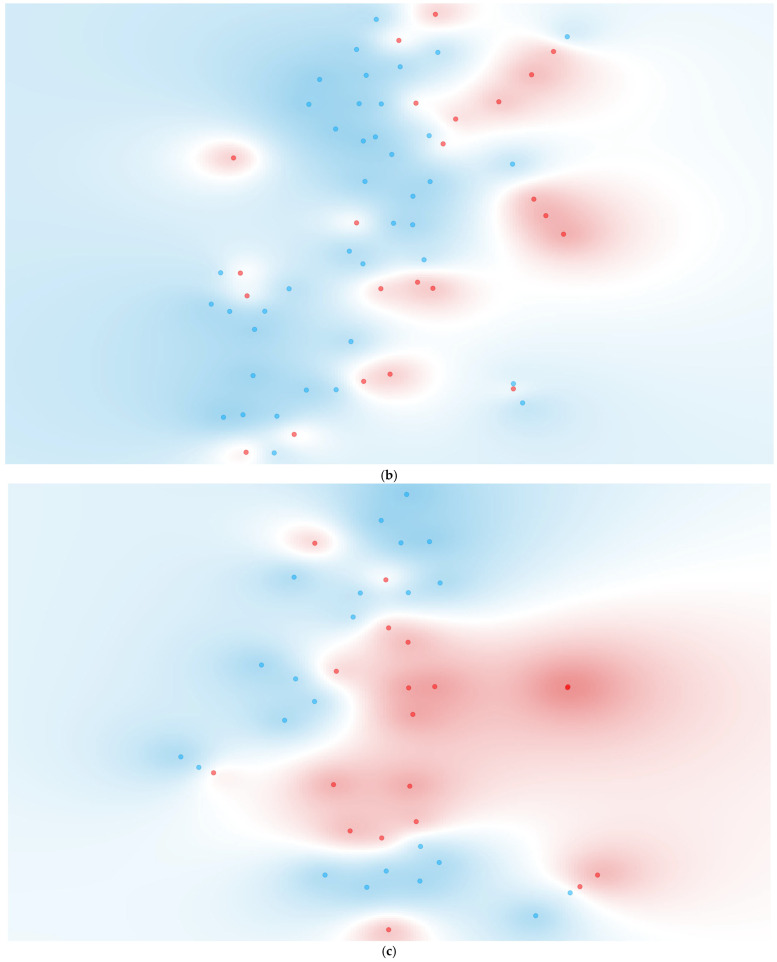

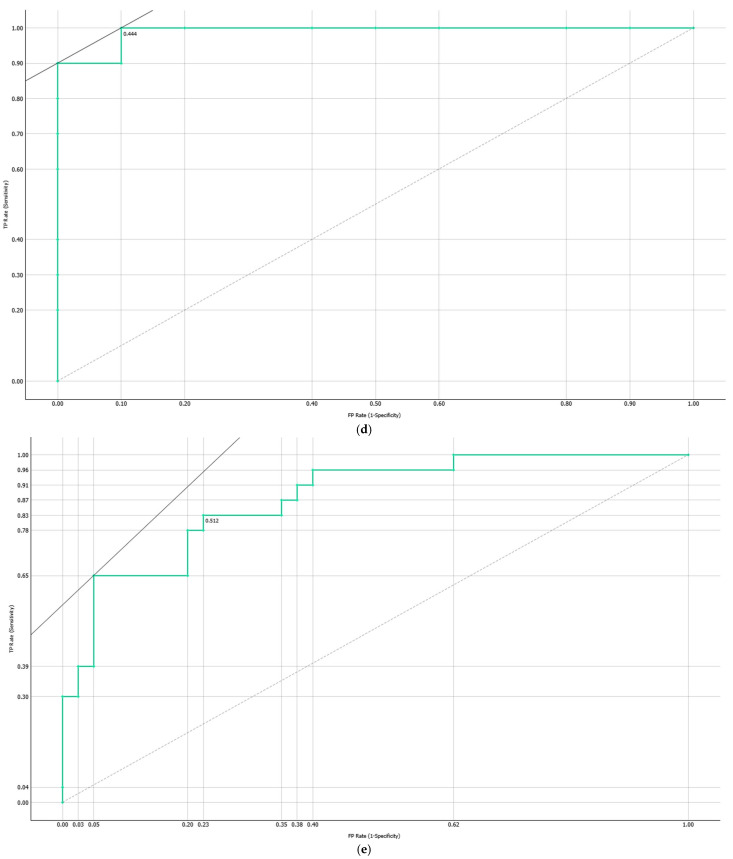

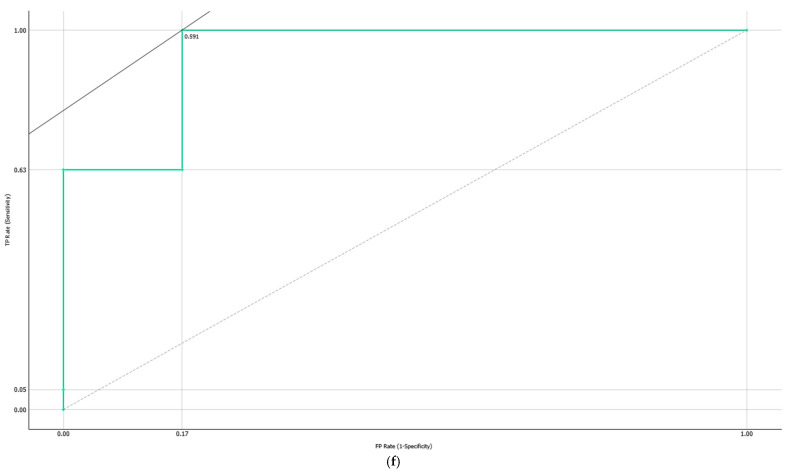
t-SNE visualization of Fast Correlation-Based Filter-ranked cytokine ratios used for Naïve Bayes classification of mortality in ICU COVID-19 patients (deceased: red, discharged: blue). Visualization for Group 1 (≤48 h) using 8 ranked cytokine ratios (**a**), Group 2 (>48 h to ≤7 days) using 6 ranked cytokine ratios (**b**), and Group 3 (>7 to ≤14 days) using 7 ranked cytokine ratios (**c**). ROC curves for the corresponding Naïve Bayes models trained with the same Fast Correlation-Based Feature-ranked features are shown for Group 1 (**d**), Group 2 (**e**), and Group 3 (**f**).

Expanding upon the initial findings from individual cytokines, this section evaluates the performance of multivariate models using the original 21 cytokines supplemented with 19,229 programmatically generated cytokine ratios, resulting in a total of 19,250 features. These ratios were derived from biologically relevant combinations of up to a sum of three pro-inflammatory cytokines in the numerator and up to a sum of three anti-inflammatory cytokines in the denominator. These complex ratios aim to represent more nuanced immunological interactions. The same machine learning models and evaluation metrics (AUC, sensitivity, and specificity) as in Section 3.4.1 were applied, with FCBF ranking used once again to refine the datasets. Results are summarized in Table 8, with corresponding t-SNE visualizations and ROC curves for the top-performing Naïve Bayes models displayed in Figure 6.

In Group 1 (≤48 h), the inclusion of complex ratios resulted in a marked performance improvement across nearly all models. Notably, the Naïve Bayes model achieved perfect AUC (1.000), with high sensitivity (0.900) and specificity (0.900). Random Forest also performed strongly with both sensitivity and specificity reaching 0.900 (AUC = 0.900). Compared to Section 3.4.1, where the best-performing model (Decision Tree) only reached 0.700 sensitivity and 0.575 AUC, the improvement is substantial. Furthermore, models that previously showed marginal utility—such as kNN and SVM—also demonstrated enhanced performance, though they did not lead overall. The t-SNE projection for this group in Figure 6a reinforces these results, showing well-separated and compact clusters of deceased and discharged patients, a considerable improvement from the diffuse and overlapping clusters in Figure 5a.

The best-performing composite ratio in Group 1—(IL-1β + IL-12p70 + MIP-1α)/(IL-13 + IL-5 + IL-21)—reflects an imbalance favoring pro-inflammatory mediators over Th2/regulatory cytokines within the first 48 h of ICU stay. While individual components of this ratio did not reach statistical significance independently in our cohort (e.g., IL-1β: *p* = 0.780, IL-5: *p* = 0.515; Table 5), their combined discriminatory power suggests a coordinated dysregulation of inflammatory pathways. This observation aligns with findings from a prospective ICU study on severe COVID-19, in which IL-1β and IL-12p70 showed modest odds ratios in univariate models (OR = 3.1 and 0.91, respectively), with corresponding *p*-values of 0.015 and 0.823. Regarding the multivariate analysis of day 1, only IL-1β retained significance (adjusted OR = 2.79, *p* = 0.035) [90]. In our dataset, IL-1β was higher in deceased patients (median = 1.25 [IQR 0.89]) than in discharged patients (median = 1.16 [IQR 0.35]; Table 6), a direction consistent with the conclusion found in [90], where elevated IL-1β was associated with increased mortality at ICU admission. It is important to note, however, that differences in assay platforms and patient populations limit direct comparison, and the external article did not provide stratified cytokine values by survival status for day 1. Nonetheless, it all points to the relevance of early IL-1β dynamics in shaping mortality risk and support the hypothesis that composite immune ratios offer enhanced resolution over single cytokine measures in the acute phase.

In the external article [90], IL-12p70 was not a significant predictor of mortality in univariate analysis, a finding also reflected in our data: at 48 h, IL-12p70 levels were higher in discharged patients (median = 5.29 [IQR 1.77]) than in the deceased group (median = 4.93 [IQR 3.39]). Notably, in their multivariate day 7 model, IL-12p70 was independently associated with reduced odds of mortality (adjusted OR = 0.23, *p* = 0.012), indicating that higher levels were linked to improved survival after accounting for other variables. This supports its inclusion in the numerator of our informative ratio, even though it did not reach statistical significance in our cohort. Notably, IL-6 and IL-8 were observed to be significantly elevated in Group 1 deceased patients (*p* = 0.002 each; Table 5), something reported in other works, such as in patients with acute renal failure, in which IL-6 and IL-8 levels at study enrollment were significantly associated with mortality [26], as well as in studies in which cytokine profiles were used as markers of disease severity in sepsis, with early mortality (48 h) being predicted for IL-8 (AUC = 0.780, *p*-value = 0.012) and IL-6 (AUC = 0.756, *p*-value = 0.015). These authors were also able to have successful results based on just IL-4, MCP-1, Granulocyte Colony-Stimulating Factor (G-CSF), and IL-1β [143], with TNF-α having been reported as predictors of mortality at 24 h and IL-1β consistently being observed to be independent predictors of ICU mortality at 48 h [144]. Regardless of these findings, their prediction capability is modest at best. Our data, however, suggests that it is the combined profile of multiple cytokines—rather than any single one—that best captures the severe inflammatory phenotype associated with impending mortality, with considerably superior metrics achieved for group 1. However, due to our retrospective sampling from outcome date rather than admission, these cytokine elevations likely reflect persistent or secondary inflammation rather than the classical early-phase cytokine storm.

In Group 2 (>48 h to ≤7 days), classification performance also improved notably. Naïve Bayes achieved the highest AUC (0.918) with a substantial sensitivity increase to 0.826 and a specificity of 0.775. This is a clear gain from Section 3.4.1, where the same model only reached 0.435 sensitivity. The Decision Tree also improved from 0.522 to 0.652 sensitivity, confirming the added value of cytokine ratios for this group. While the SVM continued to show high specificity (0.925), its sensitivity remained low (0.261), suggesting it is less suited for mortality prediction in this context. Importantly, Figure 6b shows a more structured t-SNE distribution than Figure 5b, with clusters of deceased patients emerging more distinctly, even if somewhat dispersed, likely due to the persistent class imbalance in this group.

For Group 2, the optimal ratio—(GM-CSF + IL-17A)/(IL-10 + IL-4 + IL-7)—represents a balance between inflammation and regulation during mid-stage ICU trajectories. This ratio’s components overlap with known prognostic markers during the first week: for example, IL-10 was significantly higher in our Group 2 deceased patients (median ~35 pg/mL vs. 23 pg/mL, a 52% increase; Table 6), and has been widely reported as elevated in fatal COVID-19 cases [139]. Similarly, an increase in the pro-inflammatory cytokine IL-17A, typically associated with Th17 cell activity, alongside deficient compensatory responses, mirrors patterns observed in the literature [52]. A notable parallel is the Dublin-Boston study, where a 4-day IL-6/IL-10 ratio trajectory outperformed individual cytokines in predicting 7-day outcomes—each unit increase in the IL-6/IL-10 ratio raised the odds of deterioration approximately 5.6-fold (OR = 5.62, 95% CI [3.22–9.81], *p* = 1.2 × 10^−9^) [145]. Our own complex ratio reflects a similar concept: a heightened inflammatory drive coupled with insufficient anti-inflammatory control. In essence, patients who succumb between days 3 and 7 exhibit sustained inflammation (e.g., elevated IL-17A) and high levels of regulatory cytokines (e.g., IL-10), consistent with the mixed cytokine response described in severe COVID-19. Interestingly, IL-10 levels appear to decline over time in the deceased group, both in our cohort (median ~42 pg/mL in Group 1 vs. ~35 pg/mL in Group 2) and in external findings (median ~3.21 pg/mL on day 1 vs. ~1.49 pg/mL on day 7) [90]. Other studies have similarly shown that combining IL-6 with clinical severity scores such as SOFA significantly improves predictive performance (AUC = 0.844 for IL-6 + SOFA vs. 0.776 for IL-6 alone on day 3), and that IL-8 and IL-10 levels remain consistently elevated in deceased patients [146].

Group 3 (>7 to ≤14 days) demonstrated the most transformative gains. In Section 3.4.1, performance was constrained by the survival of only one cytokine (IL-8) after FCBF, preventing t-SNE visualization and leading to inconsistent model sensitivity—SVM sensitivity notably dropped to 0.000, while the best model (Decision Tree) reached 0.684 sensitivity and 0.521 AUC.

With the inclusion of cytokine ratios in Section 3.4.2, Naïve Bayes achieved a perfect sensitivity (1.000), specificity of 0.826, and AUC of 0.930. Random Forest also achieved a strong AUC of 0.891, with balanced sensitivity (0.632) and specificity (0.870). Although SVM sensitivity increased slightly to 0.053, it remained underwhelming, reiterating the need to match algorithm choice to data characteristics. The corresponding t-SNE plot (Figure 6c), now made possible by the selection of seven features, revealed clear and well-separated groupings—an outcome unachievable in Section 3.4.1.

In Group 3, the selected ratio—(Fractalkine + IL-12p70 + IL-8)/(IL-10 + IL-13 + IL-7)—suggests that even late in the ICU course, outcomes hinge on the balance between ongoing inflammation and immune regulation. This ratio notably includes IL-8, a cytokine repeatedly shown to predict long-term mortality. In fact, a multiplex sepsis analysis identified IL-8 (alongside MCP-1) as the strongest individual predictor of 28-day mortality, surpassing other cytokines in accuracy [143]. Consistently, IL-8 levels remained elevated in our Group 3 deceased patients (median ~19 pg/mL) compared to discharged ICU patients (~11 pg/mL; Table 6), reflecting persistent hyperinflammation. At the same time, lower levels of cytokines in the ratio’s denominator (IL-10, IL-13, IL-7) in the deceased group suggest a collapse of protective immunity—for instance, IL-7, which supports lymphocyte recovery, was 24% lower in group 3 deceased (Table 6). This pattern aligns with prior observations that by week 2, patients in the discharged group often exhibit a rejuvenated immune response (e.g., higher IFN-γ and IL-12p70), whereas patients in the deceased group show immune exhaustion despite sustained inflammatory signaling. Thus, our late-stage ratio is consistent with literature findings: patients with unchecked inflammation (e.g., elevated IL-8 and Fractalkine) and insufficient compensatory responses (e.g., lower IL-7 and IL-13 relative to IL-10) are the most likely to succumb during the second week of critical illness.

This interpretation is further supported by findings from the GenIMS study, where persistently elevated IL-6 and IL-10 levels beyond the first week of hospitalization were associated with a more than 20-fold increase in 90-day mortality, and even elevation of just one of these cytokines significantly raised death risk [147]. Our Group 3 data similarly showed that IL-6 was approximately 80% higher in deceased patients (Table 6), while IL-10 showed only a modest 8% increase in the discharged group, suggesting an incomplete compensatory anti-inflammatory response. This fact is consistent with the mentioned study, in which even having one cytokine high (IL-6 or IL-10), with the other one low, was associated with a significantly increased mortality risk. Additionally, the ARDS study by Meduri et al. showed that deceased patients maintained high IL-6 and IL-1β levels from day 7 onward, while survivors showed declining levels over time [29]. This aligns with our own findings, where IL-6 remained substantially elevated in the deceased (~34 pg/mL; Table 6), while IL-1β was slightly lower (~1 pg/mL; Table 6), suggesting that in COVID-19, IL-6 may be a more dominant late-phase inflammatory driver than IL-1β.

Collectively, these results confirm that the integration of programmatically generated cytokine ratios significantly enhances the discriminatory performance of mortality prediction models across all time-based ICU groups. The gains are particularly notable in Groups 1 and 3, where performance had previously been limited. In addition, the improved t-SNE cluster separability observed in Figure 6 highlights the biological and computational relevance of the selected ratio-based features. Although not all algorithms benefit equally, underscoring the importance of model-feature compatibility, the overall improvement across the majority of models supports the inclusion of ratio-based features as a robust and interpretable extension of cytokine profiling.

**Figure 7:**
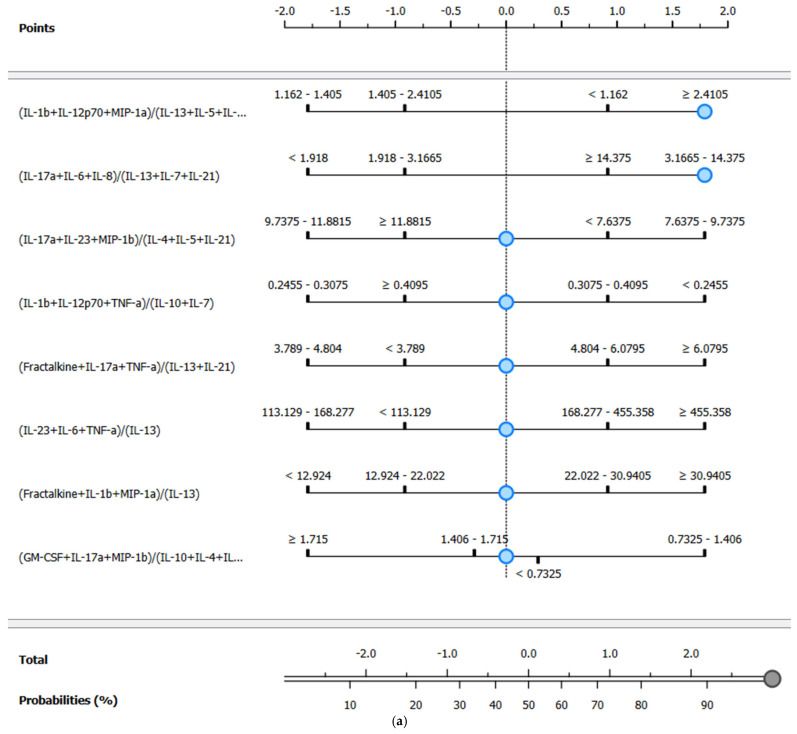

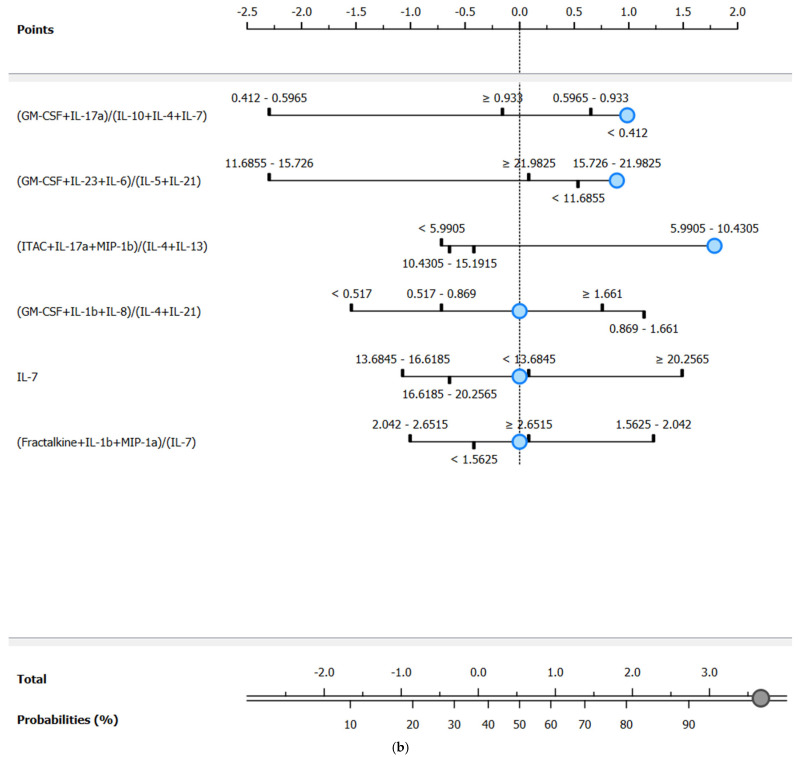

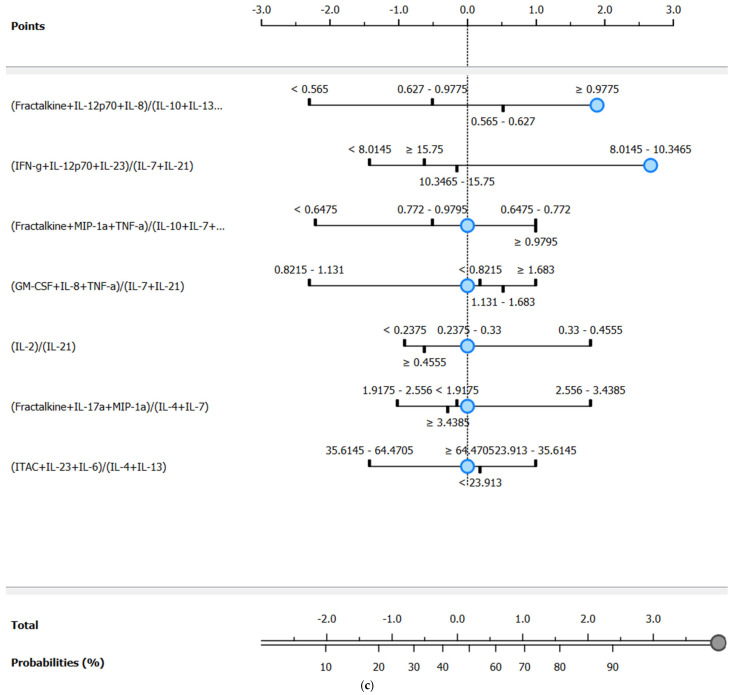
Nomograms for the ranked cytokine ratios for group 1 (**a**); group 2 (**b**), and group 3 (**c**) ICU mortality classifications.

To further interpret the contribution of key cytokine ratios to mortality prediction, nomograms (Figure 7) were generated for each of the three time-based groups, with the top features selected via FCBF. These nomograms leverage log odds ratios to visualize how specific value intervals of each complex ratio impact classification probability, with blue markers indicating reference levels corresponding to baseline classifier outputs.

In Group 1 (≤48 h), eight cytokine ratios were retained after FCBF selection. By adjusting only the top two ranked ratios, the model achieved a 95% probability of correctly identifying deceased ICU patients. Specifically, the composite ratio ((IL-1β + IL-12p70 + MIP-1α)/(IL-13 + IL-5 + IL-21)) contributed the highest number of log odds points when shifted into the ≥2.4105 interval, while the second-ranked ratio ((IL-17A + IL-6 + IL-8)/(IL-13 + IL-7 + IL-21)) added further discriminatory power within the 3.1665–14.375 interval. The remaining six ratios were held at their default reference values, indicating minimal additional predictive contribution under these conditions.

In Group 2 (>48 h to ≤7 days), six ratios were selected. If all ratios were to be kept at their default reference levels, the classification probability for a deceased ICU patient was just 37%. By shifting only the top two ratios, this probability increased to 79%.

These included the ratio ((GM-CSF + IL-17A)/(IL-10 + IL-4 + IL-7)) within the <0.412 interval, and ((GM-CSF + IL-23 + IL-6)/(IL-5 + IL-21)) within the 15.726–21.9825 range. Incorporating a third ratio, ((ITAC + IL-17A + MIP-1β)/(IL-4 + IL-13)), adjusted into the 5.9905–10.4305 interval, further raised the probability of mortality classification to 96%. These features collectively spanned immune activation, regulation, and chemotactic signaling. The remaining three ratios, including one with IL-7 as a single variable, were retained at their baseline values.

In Group 3 (>7 to ≤14 days), seven ratios were selected via FCBF, and shifting only the top two yielded a 98% prediction probability for the deceased target class. The strongest effect was observed from the ratio ((Fractalkine + IL-12p70 + IL-8)/(IL-10 + IL-13 + IL-7)), whose placement in the highest interval (≥0.9775) generated the greatest log odds shift. This was complemented by the (IFN-γ + IL-12p70 + IL-23)/(IL-7 + IL-21) ratio in the 8.0145–10.3465 range. Together, these combinations captured late-stage inflammatory imbalance while the remaining five features remained fixed at their reference positions.

Overall, the nomograms provide a transparent and intuitive framework to interpret how combinations of cytokine ratios contribute to mortality prediction. The ability to achieve high classification probabilities with minimal adjustments highlights the potency of the top-ranked ratios. Moreover, these graphical models underscore the added interpretability of multivariate cytokine profiling, which complements and strengthens machine learning predictions by offering actionable thresholds for clinical monitoring or risk stratification.

These findings justify the integration of cytokine ratios into future ICU prognostic tools and support the exploration of multivariate immune features as clinically relevant biomarkers. Section 3.5 will further contextualize these results alongside traditional severity scores and published literature.

### 3.5. Methodological Considerations and Graphical Summary of Key Findings

One fundamental consideration in complex machine learning models is, inescapably, the quality of the data feeding predictive models. In the ICU, where physiological states can change rapidly and documentation can be uneven, poorly curated records quickly lead to “garbage-in, garbage-out” predictions that no amount of extra data can fix [148]. A data-centric mindset therefore prioritizes fidelity over volume: representative cohorts, carefully verified timestamps, uniform assay methods, and transparent handling of missing data all contribute to models learning genuine physiological signals rather than artifacts. The emphasis on complex cytokine ratios in the current study follows this logic—deriving composite markers from raw measurements increases the signal-to-noise ratio, limits the impact of sporadic measurement error, and grounds the model in biology rather than in quirks of poorly maintained manual or electronic records.

Preprocessing and feature selection make this foundation usable. ICU variables arrive in different units, at irregular intervals, and with outliers that distort scale-sensitive algorithms. Rigorous cleaning, normalization, and scaling mitigate those problems, while targeted feature selection trims redundant or weak predictors that would otherwise dilute model focus [149]. In our workflow, we applied FCBF, a multivariate relevance–redundancy criterion that keeps strongly informative yet minimally overlapping variables; coupled with cytokine ratio engineering, FCBF reduced dimensionality without discarding clinically meaningful information. Other critical-care studies show similar gains when class imbalance, high dimensionality, and documentation variance are addressed early, underscoring that preprocessing often matters more than simply adding rows to a spreadsheet [150].

An additional methodological consideration relates to sample representation across time groups. Some patients contributed more than one serum sample during their ICU stay, provided these belonged to different temporal outcome groups and were collected at distinct time points. As this was not a longitudinal study, all samples were treated as metabolically independent and assigned discretely. This approach is commonly employed in ICU-based research, especially under limited cohort conditions, but we acknowledge it as a potential source of intra-patient variability.

Model evaluation must be equally deliberate. Single metrics such as accuracy or even AUC can mask weaknesses that endanger patients and compromise care, especially when adverse outcomes are rare. Current best practice advocates a multi-metric panel, using AUC for discrimination, precision–recall or F-score to address class imbalance, calibration plots for probability reliability, and decision curve analysis for bedside value, while noting that global measures such as the Brier score [151], although common [152], may offer limited clinical insight [153]. We therefore report AUC alongside sensitivity, specificity, and calibration statistics, verifying that mortality-risk gains from cytokine ratios translate into clinically actionable probability estimates. This multidimensional view prevents a model that excels at ranking patients from slipping into overconfident probability assignments or over-triggering alarms—pitfalls that frequently appear when AUC alone is considered [154].

Even with careful evaluation, no ICU model can possibly generalize to all scenarios. Demographics, treatment protocols, and laboratory equipment vary across institutions, so models trained in one hospital routinely lose performance when applied elsewhere. Even when trained on large, multi-center ICU datasets, machine learning models often fail to generalize well to new hospitals. External validation studies report AUC drops ranging from small differences, e.g., 0.04 [154] to more substantial declines of up to 0.20 in some settings or above [155], showcasing that even large-scale models can lose accuracy when applied to different patient populations. Over-reliance on public databases such as MIMIC or eICU exacerbates this issue as both originate from similar U.S. academic centers and may not reflect other health-care systems [154]. For our model, the cytokine-ratio benefit may partially reflect local assay procedures; external centers should therefore recalibrate thresholds or fine-tune weights against their baseline risk distributions before deployment.

The current study employed a targeted multiplex immunoassay to quantify cytokine levels. Like most bottom-up proteomic techniques, this method estimates protein abundance through antibody-based detection of specific epitopes, relying on antigen–antibody binding events rather than analysis of intact protein structures. As a result, it cannot distinguish between proteoforms that may differ by post-translational modifications, splice variants, or other structural variations. While this limitation constrains the scope of proteomic resolution, it does not compromise the current study’s aim, which was to investigate novel strategies for predicting ICU mortality rather than to comprehensively characterize proteoform diversity. Future studies seeking to unravel these finer molecular distinctions may benefit from integrating top-down proteomics or complementary high-resolution techniques [156,157].

**Figure 8:**
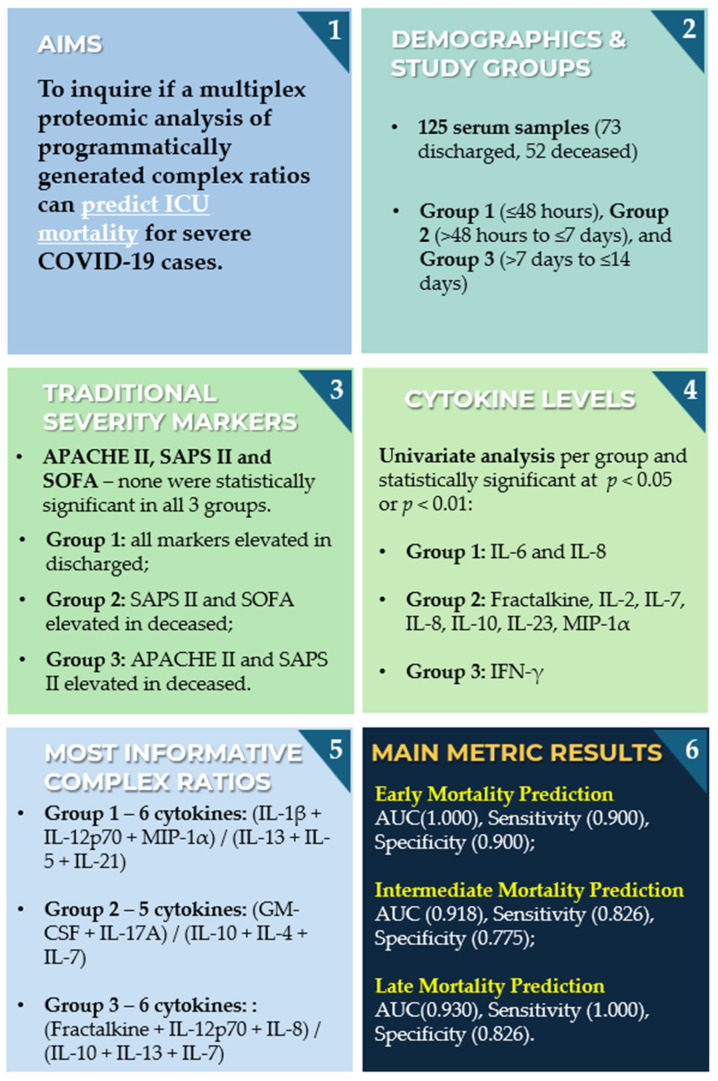
Overview of study design, methodology, and key results.

These challenges make reproducibility and adaptability especially important. Using open protocols, clear preprocessing steps, and shared code when applicable allows other researchers to repeat the work without starting from zero, turning one study’s success into progress for the wider community [158]. At the same time, models need to be flexible—retraining with a small amount of local data or adjusting thresholds can recover lost accuracy when moving to a new hospital, as seen in COVID-19 screening studies where even small site-specific tweaks made a big difference [159]. By building models with simple, modular steps, clear feature definitions, and adjustable outputs, it becomes possible to provide other institutions with a transferable framework that can be adapted to their specific clinical environments, regional demographics, and evolving ICU demands through local retraining and customization. Figure 8 provides a structured summary of the study’s main components and findings, emphasizing how multiplex cytokine profiling, complex ratio modeling, and careful machine learning practices may collectively enhance mortality prediction in ICU COVID-19 patients.

## 4. Conclusions

This study highlights the potential of multiplex cytokine profiling combined with advanced computational analyses to improve mortality prediction among ICU patients with COVID-19. Through multiplex cytokine analysis of serum samples, previously unexplored complex cytokine ratios were identified, effectively differentiating between deceased and discharged ICU patients across distinct time points of ICU stay. These biomarker profiles consistently demonstrated superior performance compared to traditional prognostic scoring systems, emphasizing the strength of cytokine-based analytical approaches in patient stratification. Unlike many existing studies based on general hospital populations or plasma samples, our study is specifically focused on ICU patients and the utilized serum, minimizing confounding factors such as coagulation-related proteins. The findings revealed clear temporal cytokine patterns: persistent or rebounding pro-inflammatory signals strongly correlated with adverse outcomes, while increased levels of immune-supportive cytokines during later stages indicated recovery. Specifically, early mortality prediction (≤48 h) reached an AUC of 1.000 using a six-cytokine ratio: (IL-1β + IL-12p70 + MIP-1α)/(IL-13 + IL-5 + IL-21). Intermediate mortality prediction (>48 h to ≤7 days) achieved an AUC of 0.918 with (GM-CSF + IL-17A)/(IL-10 + IL-4 + IL-7), and late mortality prediction (>7 to ≤14 days) resulted in an AUC of 0.930 using the ratio (Fractalkine + IL-12p70 + IL-8)/(IL-10 + IL-13 + IL-7). These findings highlight consistent involvement of IL-10, IL-13, and IL-7 in survival-associated anti-inflammatory profiles, versus elevated IL-12p70, IL-8, and Fractalkine in non-survivors. By relying on cytokines measurable through standard immunology panels, our approach offers realistic clinical applicability. Furthermore, the use of programmatically generated complex cytokine ratios, filtered through an entropy-based algorithm, enables efficient screening of tens of thousands of candidate combinations to identify high-performing, biologically plausible predictors. This not only enhances a model’s predictive capability but also opens the door to reduced assay costs by focusing on a minimal number of informative markers. Despite limitations related to its single-center design and relatively small sample size, the results suggest that the integration of multiplex cytokine profiling may serve as a valuable supplementary tool in clinical practice. Subsequent steps should include applying the workflow to non-COVID-19 ICU patients, and eventually to broader hospitalized cohorts, to assess the robustness of these biomarker strategies across disease types and treatment settings, from critical care to standard hospitalization. Future research should aim to validate these findings through multicenter studies, employing larger patient cohorts and longitudinal analyses to enhance generalizability. Integrating targeted proteomic cytokine profiling into routine clinical procedures may offer significant potential for improved risk assessment, patient management, and personalized therapeutic strategies in critical care environments.

## Figures and Tables

**Table 1:** Demographic and clinical characteristics of Group 1 (≤48 h, Early Outcome group), comprising 20 ICU samples (19 patients), 10 that correspond to patients who eventually deceased in the ICU, with the remaining 10 being discharged from the ICU. The significance level for statistical analysis comparing the two groups was set to α = 0.01 (1%).

		Deceased (*n* = 10)	Discharged (*n* = 10)	*p*-Value
Age (years), median (IQR)		68 (12)	51 (8)	0.000 (***) ^
Gender, *n* (%)	Female	4 (0.40)	0 (0.00)	0.087 +
	Male	6 (0.60)	10 (1.00)	
ECMO, *n* (%)	No	9 (0.90)	9 (0.90)	1.000 +
	Yes	1 (0.10)	1 (0.10)	
IMV, *n* (%)	No	1 (0.10)	7 (0.70)	0.020 +
	Yes	9 (0.90)	3 (0.30)	
BMI, median (IQR)		29 (8)	28 (2)	0.710 #

Statistical tests used: ^ Student’s *t*-test, # Mann–Whitney U, + Fisher’s exact test. ***: *p* < 0.001.

**Table 2:** Demographic and clinical characteristics of Group 2 (>48 h to ≤7 days, Intermediate Outcome group), comprising 63 ICU samples (57 patients), 23 that correspond to patients who eventually deceased in the ICU, with the remaining 40 being discharged from the ICU. The significance level for statistical analysis comparing the two groups was set to α = 0.01 (1%).

		Deceased (*n* = 23)	Discharged (*n* = 40)	*p*-Value
Age (years), median (IQR)		67 (8)	52 (14)	0.000 (***) ^
Gender, *n* (%)	Female	5 (0.22)	11 (0.28)	0.837 *
	Male	18 (0.78)	29 (0.72)	
ECMO, *n* (%)	No	23 (1.00)	35 (0.88)	0.149 +
	Yes	0 (0.00)	5 (0.12)	
IMV, *n* (%)	No	5 (0.22)	23 (0.57)	0.013 +
	Yes	18 (0.78)	17 (0.42)	
BMI, median (IQR)		26 (8)	29 (8)	0.922 #

Statistical tests used: ^ Student’s *t*-test, # Mann–Whitney U, * Chi-square (χ^2^), + Fisher’s exact test. *: *p* < 0.05; ***: *p* < 0.001.

**Table 3:** Demographic and clinical characteristics of Group 3 (>7 days to ≤14 days, Late Outcome group), comprising 42 ICU samples (38 patients), 19 that correspond to patients who eventually deceased in the ICU, with the remaining 23 being discharged from the ICU. The significance level for statistical analysis comparing the two groups was set to α = 0.01 (1%).

		Deceased (*n* = 19)	Discharged (*n* = 23)	*p*-Value
Age (years), median (IQR)		61 (8)	56 (10)	0.038 #
Gender, *n* (%)	Female	4 (0.21)	10 (0.43)	0.191 +
	Male	15 (0.79)	13 (0.57)	
ECMO, *n* (%)	No	14 (0.74)	23 (1.00)	0.014 +
	Yes	5 (0.26)	0 (0.00)	
IMV, *n* (%)	No	0 (0.00)	4 (0.17)	0.114 +
	Yes	19 (1.00)	19 (0.83)	
BMI, median (IQR)		26 (6)	28 (7)	0.527 #

Statistical tests used: # Mann–Whitney U, + Fisher’s exact test.

**Table 4:** Comparison of traditional severity scores, cytokines, and simple cytokine ratios according to ICU mortality outcome, stratified by time-based groups (Group 1: ≤48 h, Group 2: >48 h to ≤7 days, Group 3: >7 days to ≤14 days). Each feature is represented by its *p*-value and the group in which the elevated levels were observed (considering the median).

Group	Feature	ICU Mortality: Deceased vs. Discharged	
		*p*-Value ^1^	Elevated In
Group 1 ^3^	Traditional Severity Markers ^2^		
	APACHE II	0.277	Discharged
	SAPS II	0.125	Discharged
	SOFA	1.000	Discharged
	Cytokines		
	Fractalkine	0.094	Deceased
	GM-CSF	0.629	Discharged
	IFN-γ	0.571	Deceased
	IL-1β	0.780	Deceased
	IL-6	0.002 (**)	Deceased
	IL-8	0.002 (**)	Deceased
	IL-10	0.173	Deceased
	IL-17A	0.116	Deceased
	TNF-α	0.066	Deceased
	Simple Ratios ^6^		
	IL-1β/IL-10	0.043 (*)	Discharged
	IL-6/IL-10	0.002 (**)	Deceased
	IL-6/Lymphocytes	0.010 (*)	Deceased
	IL-10/Lymphocytes	0.040 (*)	Deceased
	Neutrophil/Lymphocytes	0.015 (*)	Deceased
	TNF-α/IL-10	0.048 (*)	Discharged
Group 2 ^4^	Traditional Severity Markers		
	APACHE II	0.825	None ^7^
	SAPS II	0.438	Deceased
	SOFA	0.316	Deceased
	Cytokines		
	Fractalkine	0.003 (**)	Deceased
	GM-CSF	0.131	Deceased
	IFN-γ	0.320	Deceased
	IL-1β	0.060	Deceased
	IL-6	0.079	Deceased
	IL-8	0.001 (**)	Deceased
	IL-10	0.015 (*)	Deceased
	IL-17A	0.176	Deceased
	TNF-α	0.102	Deceased
	Simple Ratios ^6^		
	IL-1β/IL-10	0.097	Discharged
	IL-6/IL-10	0.479	Discharged
	IL-6/Lymphocytes	0.001 (**)	Deceased
	IL-10/Lymphocytes	0.000 (***)	Deceased
	Neutrophil/Lymphocytes	0.009 (**)	Deceased
	TNF-α/IL-10	0.018 (*)	Discharged
Group 3 ^5^	Traditional Severity Markers		
	APACHE II	0.699	Deceased
	SAPS II	0.861	Deceased
	SOFA	0.755	None ^7^
	Cytokines		
	Fractalkine	0.458	Deceased
	GM-CSF	0.899	Deceased
	IFN-γ	0.001 (**)	Discharged
	IL-1β	0.639	Discharged
	IL-6	0.186	Deceased
	IL-8	0.070	Deceased
	IL-10	0.479	Discharged
	IL-17A	0.536	Discharged
	TNF-α	0.369	Discharged
	Simple Ratios ^6^		
	IL-1β/IL-10	0.719	None ^8^
	IL-6/IL-10	0.029 (*)	Deceased
	IL-6/Lymphocytes	0.313	Deceased
	IL-10/Lymphocytes	0.911	Deceased
	Neutrophil/Lymphocytes	0.617	Discharged
	TNF-α/IL-10	0.255	Deceased

^1^ *: *p* < 0.05; **: *p* < 0.01; ***: *p* < 0.001; ^2^ SOFA scores range from 0 to 24, with higher scores indicating greater organ dysfunction. SAPS II scores range from 0 to 163, and APACHE II scores range from 0 to 71; in both cases, higher scores are associated with increased mortality risk; ^3^ Deceased: 10 samples; Discharged: 10 samples; ^4^ Deceased: 23 samples; Discharged: 40 samples; ^5^ Deceased: 19 samples; Discharged: 23 samples; ^6^ Percentage of missing values for the lymphocyte-based ratios (IL-6/lymphocyte, IL-10/lymphocyte, neutrophil/lymphocyte): Group 1—Deceased = 30.0%, Discharged = 40.0%; Group 2—Deceased = 52.2%, Discharged = 42.5%; Group 3—Deceased = 57.9%, Discharged = 52.2%; ^7^ Median values equal even when rounding up to 2 or more decimal places; ^8^ Median values equal when rounding up to 2 decimal places: Deceased = 0.03 (0.034); Discharged = 0.03 (0.031).

**Table 5:** Comparison of cytokine *p*-values across ICU mortality outcome (deceased vs. discharged) for the three time-based groups (Group 1: ≤48 h, Group 2: >48 h to ≤7 days, Group 3: >7 days to ≤14 days). Cytokines with statistically significant differences (*p* ≤ 0.01) are highlighted in bold within the table and also summarized separately in Figure 3.

Cytokine	Group 1 *p*-Value	Group 2 *p*-Value	Group 3 *p*-Value
Fractalkine	0.094	**0.003** (**)	0.458
GM-CSF	0.629	0.131	0.899
IFN-γ	0.571	0.320	**0.001** (**)
IL-1β	0.780	0.060	0.639
IL-2	0.349	0.022 (*)	0.935
IL-4	0.951	0.497	0.242
IL-5	0.515	0.071	0.403
IL-6	**0.002** (**)	0.079	0.186
IL-7	0.744	0.033 (*)	0.102
IL-8	**0.002** (**)	**0.001** (**)	0.070
IL-10	0.173	0.015 (*)	0.479
IL-12p70	0.221	0.298	0.784
IL-13	0.992	0.582	0.438
IL-17A	0.116	0.176	0.536
IL-21	0.850	0.201	0.235
IL-23	0.822	0.020 (*)	0.382
ITAC	0.231	0.407	0.915
MIP-1α	0.166	0.034 (*)	0.159
MIP-1β	0.178	0.560	0.337
MIP-3α	0.048 (*)	0.186	0.543
TNF-α	0.066	0.102	0.369

*: *p* < 0.05; **: *p* < 0.01.

**Table 6:** Comparison of cytokine levels between deceased and discharged ICU COVID-19 patients across the three time-based groups. The table reports median values with interquartile ranges, absolute differences, percentage differences, and fold changes to highlight key disparities.

Group	Cytokine	Deceased Median (IQR)	Discharged Median (IQR)	Abs. Diff.	% Diff.	Fold Change
Group 1	Fractalkine	21.95 (13.53)	15.32 (7.75)	6.63	30.22	1.43
	GM-CSF	7.74 (4.87)	8.16 (2.61)	0.42	−5.42	0.95
	IFN-γ	32.54 (12.42)	30.56 (13.99)	1.98	6.07	1.06
	IL-1β	1.25 (0.89)	1.16 (0.35)	0.09	7.50	1.08
	IL-2	3.17 (1.39)	2.97 (0.59)	0.20	6.44	1.07
	IL-4	14.32 (9.22)	12.17 (4.65)	2.15	15.02	1.18
	IL-5	6.77 (3.94)	4.52 (5.21)	2.24	33.13	1.50
	IL-6	355.71 (5119.42)	1.45 (46.12)	354.26	99.59	244.56
	IL-7	17.02 (10.28)	17.64 (2.70)	0.62	−3.64	0.96
	IL-8	21.94 (937.13)	6.43 (12.38)	15.50	70.67	3.41
	IL-10	41.77 (121.49)	21.54 (37.08)	20.23	48.43	1.94
	IL-12p70	4.93 (3.39)	5.29 (1.77)	0.36	−7.32	0.93
	IL-13	2.86 (2.40)	2.59 (1.54)	0.27	9.43	1.10
	IL-17A	31.82 (27.09)	24.78 (6.93)	7.04	22.11	1.28
	IL-21	12.23 (6.79)	11.53 (2.48)	0.70	5.71	1.06
	IL-23	214.19 (164.28)	235.19 (26.45)	21.00	−9.81	0.91
	ITAC	81.96 (77.07)	59.06 (65.15)	22.90	27.94	1.39
	MIP-1α	27.47 (36.49)	20.91 (6.81)	6.56	23.87	1.31
	MIP-1β	22.15 (32.38)	18.57 (14.90)	3.59	16.19	1.19
	MIP-3α	15.39 (50.11)	12.10 (4.67)	3.29	21.39	1.27
	TNF-α	13.59 (18.18)	7.78 (6.52)	5.81	42.76	1.75
Group 2	Fractalkine	17.68 (7.33)	13.94 (5.72)	3.75	21.19	1.27
	GM-CSF	7.32 (7.62)	7.31 (4.37)	0.02	0.25	1.00
	IFN-γ	28.98 (17.36)	24.80 (18.39)	4.18	14.42	1.17
	IL-1β	1.27 (0.73)	0.99 (0.63)	0.28	21.88	1.28
	IL-2	3.37 (2.95)	2.82 (2.04)	0.55	16.36	1.20
	IL-4	13.14 (12.62)	10.09 (10.85)	3.05	23.22	1.30
	IL-5	7.45 (8.33)	4.81 (3.85)	2.64	35.46	1.55
	IL-6	14.24 (45.25)	2.66 (11.10)	11.59	81.36	5.36
	IL-7	20.43 (8.53)	15.55 (4.98)	4.87	23.86	1.31
	IL-8	23.92 (24.91)	7.14 (15.79)	16.77	70.13	3.35
	IL-10	35.02 (43.04)	23.10 (16.95)	11.91	34.02	1.52
	IL-12p70	6.01 (3.43)	4.35 (3.21)	1.66	27.64	1.38
	IL-13	3.12 (3.10)	2.87 (2.29)	0.25	7.88	1.09
	IL-17A	32.24 (34.43)	25.38 (17.40)	6.86	21.28	1.27
	IL-21	12.61 (6.87)	10.96 (6.77)	1.65	13.10	1.15
	IL-23	291.97 (227.01)	207.29 (128.71)	84.68	29.00	1.41
	ITAC	82.06 (94.83)	79.91 (76.65)	2.15	2.62	1.03
	MIP-1α	19.11 (8.15)	15.19 (11.52)	3.91	20.48	1.26
	MIP-1β	15.93 (19.02)	14.11 (20.22)	1.82	11.43	1.13
	MIP-3α	12.34 (6.93)	8.98 (7.88)	3.37	27.27	1.37
	TNF-α	10.01 (6.31)	9.30 (5.53)	0.71	7.07	1.08
Group 3	Fractalkine	15.53 (3.90)	14.07 (5.60)	1.46	9.40	1.10
	GM-CSF	7.47 (4.41)	7.10 (4.67)	0.38	5.03	1.05
	IFN-γ	24.48 (17.43)	37.15 (19.86)	12.66	−51.72	0.66
	IL-1β	1.06 (0.56)	1.19 (0.68)	0.13	−11.75	0.89
	IL-2	3.05 (1.72)	3.51 (2.42)	0.47	−15.34	0.87
	IL-4	12.65 (8.21)	11.21 (8.13)	1.44	11.37	1.13
	IL-5	6.12 (4.35)	6.20 (5.68)	0.08	−1.24	0.99
	IL-6	33.92 (153.62)	6.80 (65.22)	27.13	79.97	4.99
	IL-7	14.89 (4.71)	17.71 (3.66)	2.82	−18.93	0.84
	IL-8	18.92 (34.97)	11.24 (13.62)	7.68	40.62	1.68
	IL-10	28.52 (18.41)	30.74 (16.10)	2.21	−7.76	0.93
	IL-12p70	3.76 (4.56)	4.78 (2.84)	1.02	−26.98	0.79
	IL-13	2.45 (1.17)	2.65 (2.28)	0.19	−7.82	0.93
	IL-17A	26.85 (23.26)	29.58 (19.98)	2.73	−10.18	0.91
	IL-21	9.78 (5.38)	12.15 (7.70)	2.37	−24.18	0.81
	IL-23	215.79 (123.55)	237.36 (365.18)	21.57	−10.00	0.91
	ITAC	71.73 (123.92)	92.99 (72.44)	21.26	−29.65	0.77
	MIP-1α	24.93 (21.65)	19.84 (12.86)	5.09	20.43	1.26
	MIP-1β	15.87 (13.48)	11.07 (17.52)	4.80	30.27	1.43
	MIP-3α	12.15 (10.85)	11.26 (8.15)	0.89	7.29	1.08
	TNF-α	8.44 (6.35)	9.06 (6.51)	0.62	−7.31	0.93

Interquartile ranges (IQR).

**Table 7:** Performance of five-fold cross-validation for distinguishing between discharged and deceased in ICU COVID-19 patients, using 21 cytokines. The metrics presented correspond to deceased cases, for all 3 studied groups, obtained using various machine learning models, before and after applying Fast Correlation-Based Filter for feature selection.

Target Group *	Model	AUC	Sensitivity	Specificity
Group 1	kNN	0.800	0.500	0.800
	Naïve Bayes	0.650	0.500	0.700
	Random Forest	0.725	0.500	0.600
	SVM	0.500	0.600	0.700
	Decision Tree	0.525	0.500	0.600
Group 1 Ranked: FCBF (3 features)	kNN	0.850	0.600	0.900
	Naïve Bayes	0.775	0.500	0.600
	Random Forest	0.675	0.500	0.600
	SVM	0.850	0.300	0.400
	Decision Tree	0.575	0.700	0.600
Group 2	kNN	0.573	0.217	0.800
	Naïve Bayes	0.689	0.522	0.625
	Random Forest	0.716	0.348	0.850
	SVM	0.693	0.435	0.875
	Decision Tree	0.574	0.435	0.700
Group 2 Ranked: FCBF (2 features)	kNN	0.704	0.348	0.825
	Naïve Bayes	0.781	0.435	0.850
	Random Forest	0.646	0.391	0.800
	SVM	0.751	0.217	0.925
	Decision Tree	0.613	0.522	0.775
Group 3	kNN	0.493	0.474	0.522
	Naïve Bayes	0.244	0.316	0.217
	Random Forest	0.505	0.263	0.696
	SVM	0.367	0.158	0.565
	Decision Tree	0.371	0.474	0.435
Group 3 Ranked: FCBF (1 feature)	kNN	0.542	0.526	0.696
	Naïve Bayes	0.647	0.474	0.696
	Random Forest	0.450	0.421	0.609
	SVM	0.441	0.000	0.957
	Decision Tree	0.521	0.684	0.348

* Group 1: ≤48 h; Group 2: >48 h to ≤7 days; Group 3: >7 days to ≤14 days. K-Nearest Neighbors (kNN), Support Vector Machines (SVM). Fast Correlation-Based Filter (FCBF).

**Table 8:** Performance of five-fold cross-validation for distinguishing discharged and deceased in ICU COVID-19 patients, using 21 cytokines and 19,229 ratios. The metrics presented correspond to deceased cases, for all 3 studied groups, obtained using various machine learning models before and after applying Fast Correlation-Based Filter for feature selection.

Target Group *	Model	AUC	Sensitivity	Specificity
Group 1	kNN	0.850	0.600	0.900
	Naïve Bayes	n.a. ^1^	0.700	0.800
	Random Forest	0.800	0.500	0.800
	SVM	0.425	0.600	0.500
	Decision Tree	0.625	0.900	0.400
Group 1 Ranked: FCBF (8 features)	kNN	0.900	0.700	1.000
	Naïve Bayes	1.000	0.900	0.900
	Random Forest	0.900	0.900	0.900
	SVM	0.450	0.700	0.800
	Decision Tree	0.800	0.800	0.500
Group 2	kNN	0.637	0.304	0.875
	Naïve Bayes	n.a. ^1^	0.652	0.500
	Random Forest	0.622	0.435	0.675
	SVM	0.420	0.043	0.950
	Decision Tree	0.619	0.435	0.750
Group 2 Ranked: FCBF (6 features)	kNN	0.722	0.261	0.925
	Naïve Bayes	0.918	0.826	0.775
	Random Forest	0.774	0.522	0.800
	SVM	0.750	0.261	0.925
	Decision Tree	0.666	0.652	0.700
Group 3	kNN	0.472	0.579	0.435
	Naïve Bayes	n.a. ^1^	0.526	0.522
	Random Forest	0.570	0.316	0.609
	SVM	0.537	0.000	0.957
	Decision Tree	0.533	0.526	0.565
Group 3 Ranked: FCBF (7 features)	kNN	0.593	0.421	0.696
	Naïve Bayes	0.930	1.000	0.826
	Random Forest	0.891	0.632	0.870
	SVM	0.384	0.053	0.826
	Decision Tree	0.662	0.632	0.696

* Group 1: ≤48 h; Group 2: >48 h to ≤7 days; Group 3: >7 days to ≤14 days. ^1^ Some scores could not be computed (n.a.—not available). K-Nearest Neighbors (kNN), Support Vector Machines (SVM). Fast Correlation-Based Filter (FCBF).

## Data Availability

The data presented in this study are not publicly available, due to privacy or ethical restrictions, and due to the ongoing nature of the study.

## Abbreviations

The following abbreviations are used in this manuscript:

APACHE II	Acute Physiology and Chronic Health Evaluation II
ARDS	Acute Respiratory Distress Syndrome
AUC	Area Under the Curve
BMI	Body Mass Index
COVID-19	Coronavirus Disease 2019
c-SAPS3	customized Simplified Acute Physiology Score 3
CRP	C-Reactive Protein
ECMO	Extracorporeal Membrane Oxygenation
FC	Fold change
FCBF	Fast Correlation-Based Filter
GCS	Glasgow Coma Scale
G-CSF	Granulocyte Colony-Stimulating Factor
GM-CSF	Granulocyte-Macrophage Colony-Stimulating Factor
ICU	Intensive Care Unit
IFN-γ	Interferon-gamma
IL-1β	Interleukin-1 beta
IL-2	Interleukin-2
IL-4	Interleukin-4
IL-5	Interleukin-5
IL-6	Interleukin-6
IL-7	Interleukin-7
IL-8	Interleukin-8
IL-10	Interleukin-10
IL-12p70	Interleukin-12p70
IL-13	Interleukin-13
IL-17A	Interleukin-17A
IL-21	Interleukin-21
IL-23	Interleukin-23
IMV	Invasive Mechanical Ventilation
IQR	Interquartile Range
ITAC	Interferon-inducible T cell Alpha Chemoattractant
KNN	k-Nearest Neighbors
MIP-1α	Macrophage Inflammatory Protein-1 alpha
MIP-1β	Macrophage Inflammatory Protein-1 beta
MIP-3α	Macrophage Inflammatory Protein-3 alpha
NLR	Neutrophil-to-Lymphocyte Ratio
OASIS	Oxford Acute Severity of Illness Score
PCA	Principal Component Analysis
PREMO	Predictive Models of COVID-19 Outcomes for Higher-risk Patients Towards a Precision Medicine
RT-PCR	Real-Time Polymerase Chain Reaction tests
SAPS	Simplified Acute Physiology Score
SARS-CoV-2	Severe Acute Respiratory Syndrome coronavirus 2
SICULA	Super ICU Learner Algorithm
SIRS	Systemic Inflammatory Response Syndrome
SOFA	Sequential Organ Failure Assessment
SVM	Support Vector Machine
t-SNE	t-distributed Stochastic Neighbor Embedding
TNF-α	Tumor Necrosis Factor-alpha
